# Three-Dimensional Printing in Paediatrics: Innovative Technology for Manufacturing Patient-Centred Drug Delivery Systems

**DOI:** 10.3390/pharmaceutics17111364

**Published:** 2025-10-22

**Authors:** Nadine Couți, Sonia Iurian, Alina Porfire, Tibor Casian, Rareș Iovanov, Ioan Tomuță

**Affiliations:** Department of Pharmaceutical Technology and Biopharmacy, Faculty of Pharmacy, “Iuliu Haţieganu” University of Medicine and Pharmacy, 41 V. Babes Street, 400012 Cluj-Napoca, Romania; nadine.couti@elearn.umfcluj.ro (N.C.); aporfire@umfcluj.ro (A.P.); casian.tibor@umfcluj.ro (T.C.); riovanov@umfcluj.ro (R.I.); tomutaioan@umfcluj.ro (I.T.)

**Keywords:** paediatric formulations, child-appropriate medicines, printlets, chewable dosage forms, fused deposition modelling, direct powder extrusion, semi-solid extrusion

## Abstract

Additive manufacturing can be regarded as a game-changing approach for paediatric drug development, as children have special drug-related requirements which are rarely met by conventional technologies. Traditional dosage forms have considerable drawbacks, among them dose, excipient safety, and taste issues, which can be resolved by using three-dimensional (3D) printing. Ease of swallowing and an appealing design are among the improvements brought forth by 3D printing techniques. Techniques that have been thoroughly researched in the paediatric field include hot-melt extrusion (HME) coupled with fused deposition modelling (FDM), direct powder extrusion (DPE) and semisolid extrusion (SSE) 3D printing. Selective Laser Sintering (SLS) 3D bioprinting and binder-jet (BJ) 3D printing are other less known but highly useful techniques. A number of studies focus on significant subjects for the paediatric medicine domain, such as the acceptability of the produced formulations, the size of tablets, the design, the concealment of bitter API flavour, and the stability of the dosage forms. The 3D-printed oral formulations are varied: conventional-sized tablets, miniaturised tablets, chewable tablets, and orodispersible films or tablets. Most of the drugs used in the presented studies are essential medicines for children, for which commercial products with flexible doses and age-appropriate characteristics are often lacking. The practical implications of currently published studies and future directions for paediatric pharmaceutical 3D printing are described. Although there is a substantial amount of technical and in vitro data as well as paediatric engagement work on this subject, its translation into clinical practice is still limited. The clinical efficacy of 3D-printed dosage forms has to be further researched, since only a few studies have targeted this aspect.

## 1. Introduction

In recent years, additive manufacturing has been one of the most challenging topics in drug research, offering feasible solutions to medicine accessibility issues and personalised therapies. Of the proposed applications, one that promises a major positive impact on society and aligns with the new World Health Organization (WHO) initiatives is the on-demand manufacturing of paediatric drugs. The dosage and administration requirements for paediatric patients differ from those of other population subgroups, because children are in continuous development. Different drug doses are needed considering the individual’s age and weight. It is therefore desirable to have age-appropriate oral medication delivery systems that are specially designed to cater to the demands of the paediatric population [1]. The most important requirements for dosage forms designed for this population include dose flexibility, size or volume compliance, ease of handling, ease of administration, minimum influence on lifestyle, acceptable flavour and aspect, and limited frequency of administration. For the patient’s safety, the following factors are vital: tolerability, limited excipient count, stability during use and shelf life, and low risk of dosage error [2]. In light of the present emphasis on examining the effectiveness of pharmaceuticals in paediatric patients, it is critical to also consider the factors that influence paediatric patients’ drug compliance, as for several paediatric diseases, there is evidence linking low adherence to negative health effects [3]. Taste has a major role in determining palatability, which is problematic because many active pharmaceutical ingredients (APIs) on the market and in development have unpleasant tastes. Swallowing difficulty can be a problem in the paediatric population, which makes it harder to disguise flavour when using oral dosage forms, including liquid formulations and orodispersible or chewable tablets. Given postnatal sensory system maturation and distinct taste reactions from adults, children are not simply smaller versions of adults. They have an increased preference for sugary foods and an aversion to products with a bitter flavour. Furthermore, children of different ages metabolise excipients and APIs in different ways [4]. Consequently, excipient selection becomes more challenging, since some excipients may not be suitable or their concentrations should be limited. In addition to safety concerns, some excipients affect the pace at which the concurrently administrated drugs are absorbed and eliminated, which can lead to pharmacologic interactions [5]. Moreover, each child’s and family’s daily routine and lifestyle should be considered while designing the therapeutic regimen, including the frequency and time of administration [3].

Even though a sizeable portion of the population cannot swallow tablets or capsules, these dosage forms are still the industry standard. The large size of the tablets or capsules, poor palatability, and inappropriate dosing strength of these oral formulations can be causes for medication failure. The target population’s swallowing ability, body size, and age are the main factors that determine the design requirements for oral formulations. Since the target population’s age ranges from birth to eight or ten years old, establishing correct design requirements can be challenging, as a single dosage form is not suitable to cover such a broad range. The conventional manufacturing methods offer some child-adapted dosage forms, such as mini-tablets, sprinkles, pellets, and orodispersible formulations [6].

Owing to its ability to adjust the treatment to patients’ needs, 3D printing is a rapidly growing field of research in the pharmaceutical sector and it can bring several advantages compared to conventional techniques [7]. In a therapeutic context, one of the benefits provided by 3D printing is on-demand dispensing, which could allow for faster discharge times while improving patient access to medications, particularly for those that call for specialised manufacturing or last-minute preparations. Therefore, in situations when time or resources are limited, 3D printing can quickly produce one-off dosages and obtain tailored doses [8]. As far as the cost is concerned, a framework providing insights into 3D printing manufacturing costs demonstrated that the cost of one 3D-printed hydrocortisone tablet would be, at most, 3 euros [9]. Another advantage is that based on the patient’s age and weight, customised doses can be provided. The release profile, size, and design of the printed product can also be tailored with the help of 3D printing [10]. Also, 3D-printed dosage forms can enhance drug acceptance. In addition to that, polypills can be created using 3D printing techniques, which can greatly reduce the number of pills one patient has to take [11].

In recent years, a variety of 3D printing technologies have been used, out of which fused deposition modelling (FDM), direct powder extrusion (DPE), and semi-solid extrusion (SSE) were the most used widespread in terms of paediatric dosage forms. A number of review articles focusing on 3D printing methods for paediatric medicines, in general, or on just one specific method, have been published throughout the years. A mini review debated the feasibility of 3D printing dosage forms for children and found that considerable challenges remain [12]. Some of these challenges have been overcome in the last few years and will comprise the subject of this article. Another review concentrated on listing the advantages and challenges of 3D printing for the paediatric domain, noted a few experimental studies, and gave a professional opinion based on the author’s work as a paediatric pharmacist, which is worth reading [10]. Regarding the clinical setting, some improvements have been made since its publication, which will be further presented here. An additional published review on this subject made pertinent remarks about the accuracy of dosing flexibility, gave examples of studies comparing 3D printing with conventional techniques, addressed the palatability of 3D printed dosage forms, but did not discuss at length the medicinal products prepared with each 3D printing technique, which is something that will be displayed in the following subchapters [13]. A comparison between 3D printing, microfluidic platform, and prilling was made, but, for 3D printing, the focus was on DPE and pressure-assisted microsyringe (PAM), while the other techniques were only generally presented [14]. A distinct article mentioned companies that develop 3D printers with a pharmaceutical aim, composed an overview of paediatric formulations prepared with HME and FDM, and provided a landscape of the market at the time of publication [15]. A review based on FDM 3D printed dosage forms for children discussed HME and FDM at length in terms of excipients, filament, dosage form characterisation, and technological aspects of the FDM 3D printers [16]. In comparison, this review outlines paediatric formulations prepared with each main 3D printing technique. Another review article based on paediatric formulations has been published, but its focus was on the advantages and disadvantages of 3D printing, in general, in the pharmaceutical and medical domain, as well as specifically for paediatric patients, listing only a few of the 3D-printed preparations for children [17]. Lastly, an additional review concentrated on medical applications of 3D printing as well as various 3D-printed oral dosage forms categories for children [18]. Since new studies focusing on paediatric 3D printing are released at a rapid pace, and recent clinical advancements have been made in this area, there is a significant need for an overview of this subject. This is why the present literature study aims to bring forth the shortcomings of commonly manufactured paediatric dosage forms and to review and present the advances made in the 3D printing paediatric domain, focusing on the various dosage forms created by researchers and the most important excipients used in these studies.

## 2. Shortcomings of Medicinal Products for Paediatric Use

The paediatric population has specific needs that must be met when formulating dosage forms. Age-appropriate oral formulations should satisfy all the quality standards of traditional pharmaceutical products in addition to particular patient needs, such as greater swallowing ease and dose flexibility. The pharmacokinetic and pharmacodynamic characteristics of a drug are highly dependent on a child’s developmental stage, hence dose flexibility is necessary to meet the needs of various age groups [1]. These pharmacokinetic variations include differences in absorption because of stomach emptying time and gastric pH, and differences in drug distribution because of low plasma protein concentrations and a higher body water content. Because metabolic mechanisms are frequently underdeveloped at birth, drugs for which metabolism is an important elimination mechanism may have a longer half-life and lower clearance. Neonates also have lower renal excretion because of their immature tubular secretion, glomerular filtration, and reabsorption [19].

In addition to taking into account many aspects such as taste, appearance, palatability, and ease of swallowing, an ideal paediatric formulation also has to satisfy a number of other needs, such as those of patients, caregivers, manufacturers, and healthcare professionals [20]. The current conventional dosage forms available on the market are predominantly non-customizable and can be improper for children, which can lead to serious side effects. In addition to that, paediatric patients often undergo treatment with medicinal products intended for adult use, because no suitable alternative is marketed. In this case, in order to provide children with appropriate doses, caregivers and healthcare professionals commonly need to break pills or make suspensions by breaking and pulverising the tablets [21]. A study by Binson et al. demonstrated the fact that manipulations of off-label tablets by splitting resulted in severe underdosing [22].

In the case of oral administration of paediatric dosage forms, the available formulations are solutions, syrups, suspensions, powders, capsules, granules, tablets, ODTs, chewable or orodispersible tablets, and orodispersible films (ODFs) [23]. Nasal and otic drug delivery methods do not differ from adult formulations because children’s anatomy of the nose and ear is similar to adults’ anatomy; thus, adult products are considered effective for children. Akin to nasal and otic drug delivery, pulmonary-targeted delivery for children makes use of adult formulations with the addition of a small infant or child-sized mask instead of the adult-sized. Numerous ocular formulations include drops, ointments, gels, and inserts. The most common forms of delivery for rectal preparations include creams, ointments, suppositories, foams, sprays, and enemas [24]. One of the most reliable therapeutic options in hospitals and emergency rooms is the parenteral route, particularly in situations where the oral route is impractical, such as for neonates and critically ill children [25]. Another route of administration explored for children’s treatments is the transdermal route. It relies on the application of transdermal patches as an alternative to the oral route, fit for patients who are unable to swallow or unconscious [26]. All these dosage forms have specific shortcomings, and some of these shortcomings can be resolved through the use of 3D printing, hence this will be discussed in the next chapters of the article. The most frequently cited drawbacks of currently used paediatric dosage forms, categorised by route of administration, are summarised in Table 1.

**Figure 1:**
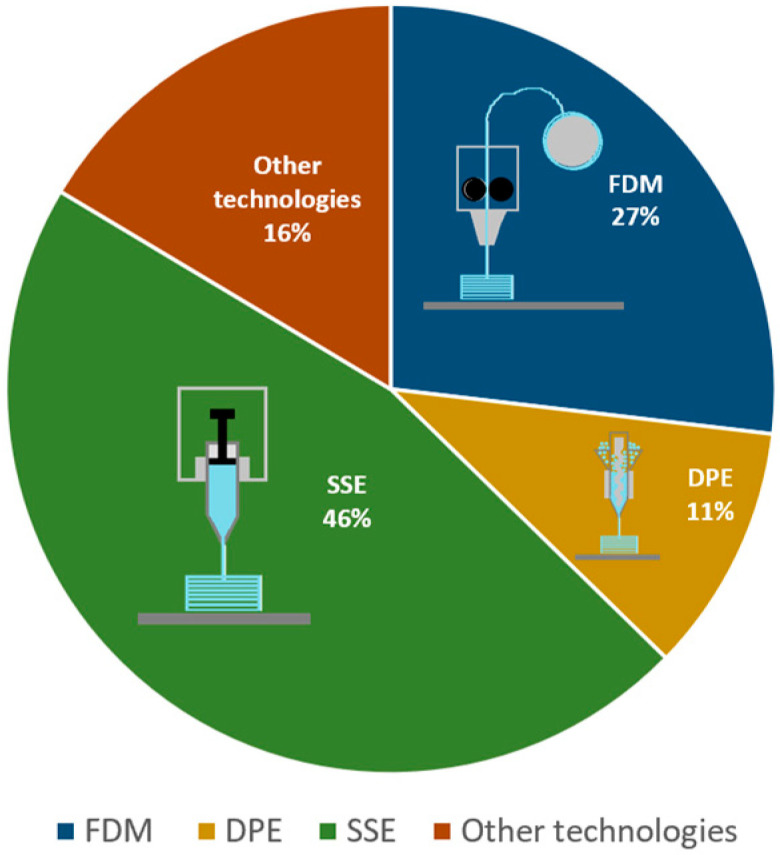
Pie chart illustrating the percentages of the most utilised 3D printing techniques found in published articles for the preparation of paediatric formulations: SSE, FDM, and DPE, and other technologies.

One of the innovative techniques that can resolve some of the issues of medicinal products currently used for paediatric patients is 3D printing; thus, in the next subchapters, the most relevant studies regarding the 3D printing of paediatric formulations are compiled. In addition to that, in Figure 1, the percentage of studies producing 3D-printed dosage forms through the most utilised techniques and diagrams of the types of equipment are shown.

## 3. Principles and Advances in HME and FDM

### 3.1. Technology

The HME process involves processing the drug and excipients initially in a powder form into an extruder with revolving screw elements at temperatures that are frequently higher than the melting temperature (Tm) and glass transition temperature (Tg) of polymers [37]. The melting and further extrusion of the molten mixture of API, polymers, and plasticizers leads to filaments that act as intermediate products for 3DP. The development of pharmaceutical products benefits from many advantages offered by HME, among which is the solvent-free process, which eliminates the need for additional drying steps. Extrusion-based FDM additive manufacturing involves dispersing the material in successive layers across the build plate after it emerges from the nozzle [7]. This technique makes it possible to customise drug therapy by having better control over API dose, dosage form shape and design, drug release, and reproducibility [38].

**Figure 2:**
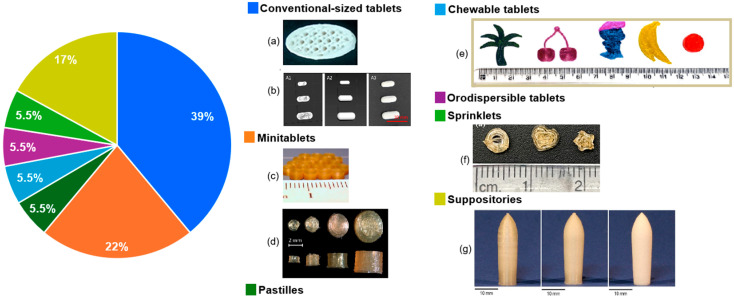
The distribution of papers reporting paediatric medicines manufactured by FDM 3D printing expressed as percentages as a function of dosage forms: conventional-sized (**a**) [39], (**b**) [40]; minitablets (**c**) [41], (**d**) [42]; pastilles; chewable tablets (**e**) [43]; orodispersible films; sprinklets (**f**) [44]; and suppositories (**g**) [45].

Several studies introduce HME and FDM as suitable techniques for the manufacture of paediatric 3D-printed dosage forms, the distribution of these can be seen in Figure 2. The advantages of HME and FDM include the improvement of patient compliance by printing specific child-friendly designs, the possibility of dose and size adjustment, and taste-masking of bitter APIs. 

### 3.2. APIs

As the therapeutic needs of the paediatric population are diverse, every 2 years, WHO issues the List of Essential Medicines for Children. Therefore, many studies have focused on proposing appropriate, child-friendly formulations for the drugs included in this list [46].

Medicines such as caffeine [39,42,47], amiodarone hydrochloride [48], propranolol hydrochloride [42], celecoxib [44], griseofulvin [41], hydrocortisone [40,49], baclofen [50], indomethacin [51], diphenhydramine hydrochloride [43], tinidazole [52], aripiprazole [53], sodium valproate [54], esomeprazole, and ondansetron [55] have so far been included in printed oral dosage forms obtained by FDM. Prednisolone sodium phosphate [45], artesunate [56], and cannabidiol [57] were used as model drugs for rectal dosage forms.

The safety and effectiveness of pharmaceutical compounds are greatly influenced by their physicochemical characteristics. Poor molecular qualities can be altered or rectified by having a fundamental understanding of physicochemical properties. In order to obtain specific information about mixtures, pre-formulation studies are a crucial step in any pharmaceutical development process. They are important for serving as reference data for additional proof-of-concept research [58]. Pre-formulation studies aim to gather information on flow characteristics and thermal behaviour, which are important during the HME step, and on the physical state of the API and excipients throughout the preparation stages. For example, researchers assessed the feasibility of utilising raw materials to create 3D-printed dosage forms based on two common APIs: esomeprazole and ondansetron. The raw materials with the best flow characteristics were found to be PVP K17 and K90. The presence of an amorphous structure was determined for PVP K17, PVP K25, PVP K90, and PVA Mw30. Blends of PVP and ondansetron showed changes in the API thermal behaviour, which pointed to PVA Mw 30’s adequacy as an excipient for ondansetron extrudates. As for esomeprazole, the study concluded that the PVPs (K17, K25, and K90) are all fit for HME [55].

### 3.3. Dose and Dose Flexibility

The reported unit doses are rather small, usually adapted to the needs of the target population, between 2 mg [49] and 25 mg [51], with the exception of one study where researchers managed to print a wide range of doses from 18 to 247 mg valproate sodium [54]. However, the dose choice must consider the filament loading capacity, which is usually small, from 1% to 20% [41], limiting the incremental options, as the addition of an API can impact quality and further processability. For instance, a study showed that a caffeine ratio increase from 5% to 20% determined poor processability as filaments became highly brittle [39]. While generally, higher drug concentrations lead to better drug recovery and purity results, a study revealed that a load of 15% hydrocortisone produced filaments that were incompatible with FDM 3D printing, which was credited to the compound’s plasticising action [40].

### 3.4. Excipients

One of the most important aspects of a paediatric medication’s development is the selection of appropriate excipients. Special safety precautions must be taken before adding any excipient to paediatric preparations, even for the ones typically approved for adult medications or found in approved paediatric medications, as children of different ages may be exposed to excipients differently than adults [59]. The WHO guideline on the pharmaceutical development of paediatric medicines has appointed some factors to consider when choosing excipients. The most important considerations are the safety profile for different age groups, the daily dose of the excipient, and the regulatory status for the target population [60]. Useful information can be found in the “Safety and Toxicity of Excipients for Paediatrics” (STEP) database [61,62] where some researchers check the excipient safety and appropriateness for administration to children [39]. Others rely on the Generally Recognised as Safe (GRAS) status when selecting proper excipients [55].

Filament-forming polymers represent the main constituents of 3D-printed dosage forms, with a high impact on both processability and the characteristics of the finished dosage forms. Cellulose-based polymers such as hydroxypropylcellulose (hyprolose, HPC) [39,41,42,43,45,47], hydroxypropylmethylcellulose (hypromellose, HPMC) [42,49], and hypromellose acetate succinate (HPMC AS) [51] have often been used for both immediate and prolonged release. HPC was used as the main polymer for the extrusion of an immediate release product with caffeine as an API [47].

A thorough selection of the polymer must be made, as it influences not only the printability, but also the dissolution behaviour. For instance, when two compounds, both classified in Biopharmaceutical Classification System (BCS) class I, were tested in combination with HPMC or HPC, propranolol hydrochloride was released more quickly when HPMC was used as the carrier polymer. In the HPC tablets, the two APIs demonstrated similar release profiles [42].

Additional water-soluble polymers tested in conjunction with HPC included vinyl-based polymers: poly-(vinyl pyrrolidone-vinyl acetate) copolymer (Kollidon^®^ VA64) and poly-(vinyl alcohol-polyethylene glycol) graft copolymer (Kollicoat^®^ IR) [39]. Kollidon VA64 and Kollicoat IR were mentioned for their rapid release properties when mixed with other matrix-forming polymers (HPC, HPMC) because of low printability when used on their own [39,49,63]. On the contrary, polyvinylpyrrolidone (PVP) types K17, K25, and K90 were used as single matrix-forming polymers in a study that used esomeprazole as its API [55]. In one study, the water-soluble poly(2-ethyl-2-oxazoline) (Aquazol^®^ 500) was used as the main polymer during HME [44]. Kollidon 25, a solubility enhancer, was used as a matrix-forming polymer in another study [52].

Poly(vinyl) alcohol (PVA), a polyhydroxy polymer, is one of the most widely used filament-forming polymers in HME, combined with FDM 3DP [64]. Parteck^®^ MXP is one of the pharmaceutical grade PVA polymers that has been used in studies aimed at paediatric formulations. It has an elevated degradation temperature, over 250 °C, expanding the application range of APIs in HME. Extrudates containing this inert polymer were stable across various scenarios, no recrystallization or degradation was shown in the case of cold, room temperature, or expedited conditions [65].

Eudragit^®^ E PO, a butyl methacrylate, dimethylaminoethyl methacrylate, methyl methacrylate copolymer, was also used as a filament-forming excipient [40,66]. It enhanced the solubility of moderately soluble drugs in amorphous solid dispersions and had special protective qualities for flavour masking, moisture protection, and shielding from deterioration. Other useful functions for paediatric dosage forms were augmenting the swallowability and masking unpleasant odours [67]. Wang et al. successfully prepared doughnut-shaped tablets containing HPC LF as a matrix-forming polymer, using HPMC K4M for a sustained release and Eudragit E PO for its taste-masking properties. However, the increase in Eudragit E PO concentrations led to a decrease in filament printability, which limited its use to 5% [47] in the presence of an API, while ratios of 45% were successfully used in placebo formulations [66].

Water soluble polyethylene oxide (PEO) was also used in FDM printing, leading to good mechanical properties in filaments at temperatures below 100 °C [48,54,68].

Polymers (HPC) are sometimes mixed with lipid-based excipients, like Gelucire^®^ 48/16 [43], which is known to improve the oral bioavailability and water solubility of poorly soluble APIs. Hydrophobic and lipophobic active substances become more soluble when used with Gelucire^®^ due to its high Hydrophilic–Lipophilic Balance (HLB), and it is most useful for drugs with a low logP. It can be used for melt operations, as it has a melting point of 48 °C [69].

Plasticizers are usually required to improve processability and product quality. Their addition often results in a reduction in processing torque and makes extrusion at a lower temperature easier [50]. The most commonly used plasticizers in the reviewed studies were mannitol, sorbitol, xylitol, polyethylene glycol (PEG) 6000, PEG 35000, and triethyl citrate, at ratios comprised between 2% and 48% [39,40,41,42,43,45,47,48,49,50,51,53,54,56,66,70]. Their high water solubility showed a positive impact on immediate release formulations. Sorbitol is known as the plasticizer of choice for fast disintegration [48]. The addition of mannitol as a pore-forming excipient can boost the rate of drug release. A group of authors proposed that formulations with a small amount of mannitol might be used for prolonged release, while those using high mannitol concentrations were fit for immediate release products [45]. The plasticizing effects of mannitol allows high API load (30 mg/unit) and improved filament printability; however, one study reported that the mannitol-containing filaments were more brittle than non-mannitol-containing filaments [45]. In one study, by adding PEG 6000, the viscosity increased, but the plasticiser migrated, acting as a lubricant and leading to a torque decrease and uncontrollable filament diameter. In contrast, by adding PEG 35000 with longer polymer chains, the migration was hindered and the HME process resulted in suitable filaments [54].

Pore-forming excipients were sometimes used to grant quick dissolution, such as maltodextrin, PEG 4000 and dibasic calcium phosphate [39]. Flow regulators such as anhydrous silica [48], sodium stearyl fumarate [40] and fumed silica [42] were added to improve the extrusion process. Surfactants such as D-alpha tocopheryl polyethylene glycol 1000 succinate (Kolliphor^®^ TPGS) and Sophorolipids (REWOFERM^®^ SL or SPL) were chosen in one study to enhance the filaments’ mechanical properties to guarantee effective printing, in addition to lowering the polymeric blends’ melt viscosity and printing temperature [44].

TiO_2_ was mentioned in several articles for its use as a colouring agent sometimes to increase the resemblance between the 3D printed dosage forms and the conventional tablets, coated with Opadry to improve their cosmetic appearance [66].

Product properties do not depend only on the formulation, but also on the HME and 3D printing parameters. In this regard, a study showed that the dissolution rate of drugs might be affected by variations of only 10 °C in 3D printing temperature, irrespective of the infill density [39]. Additionally, temperature seems to have an impact on unit weight variation, since Palekar et al. demonstrated that printing at a specific temperature (190 °C) leads to more even weights and regular surfaces [50]. Moreover, printing paediatric medicines often involves the storage of powder mixtures or intermediate products such as filaments until the printing process. Roulon et al. investigated the effect of different storage conditions on the mechanical properties of filaments and revealed that the elastic properties were not changed, in contrast with stiffness [48].

### 3.5. Dosage Forms and Acceptability

FDM showed high versatility in producing a wide array of dosage forms, summarised in the pie chart included in Figure 1. Most 3D-printed paediatric drugs were immediate-release dosage forms, with sizes, shapes, and dosages adapted to the children’s needs. Most of the tablets were cylindrical or oval shaped, with flat [39] or convex surfaces [66]. The reported sizes varied between 6 [66] and 19 mm in diameter or length. The designs were conceived according to the desired release. For instance, honeycomb geometry was selected as the most appropriate for the rapid release of caffeine. All formulations of tablets met the requirements for either very rapid release (dissolution exceeding 85% after 15 min) or rapid release (dissolution exceeding 85% after 30 min) [39]. Moreover, Wang et al. successfully prepared doughnut-shaped tablets containing caffeine citrate. The purpose of the doughnut shape was to have a higher surface area to volume ratio than typical circular tablets and to appeal to children [47]. The waffle design was used for mini tablets to satisfy the needs of the paediatric population, in terms of shape and appeal, as well as the standards for immediate release formulations, while the colour was improved using a dye [49]. A grid pattern was also used to develop oral dosage forms [54]. Some researchers have also considered the infill pattern design used in mini-caplet printing and showed that disintegration behaviour was impacted. Apparently, disintegration times increased in the following order: sharkfill, linear, hexagonal, and diamond [50]. Other studies aimed to highlight the flexible design advantage of the FDM 3D printing technique through the development of formulations that resemble sweets, inspired by Starmix^®^ candies. The size of the printed tablets was about 10 to 20 mm, depending on the design: bear, heart, lion, etc. Most importantly, given that the Starmix products effectively conceal taste due to the interactions between the drug and the excipients, 3D-printed dosage forms could be used in paediatric applications to improve palatability [51]. Moreover, one group of researchers established shape accuracy by 3D printing “sprinklets”, small-sized tablets with a 2 mm height and various designs: doughnut, heart, and star [44].

To ensure good treatment adherence, and the efficacy and safety of a therapy, product acceptability in paediatric patients is an essential requirement [71]. When their preferences were inquired about, children reported size as the most important factor where oral administration of solid dosage forms is concerned, followed by taste, texture, and smell [66]. Therefore, researchers envisaged size-reduction as a strategy to adapt dosage forms to the paediatric population. Mini-tablets, prescribed as single or multiple dosage units, are age-appropriate oral dosage forms for special groups of patients, recognised for their swallowability, which is a fundamental feature that endorses their use in the paediatrics domain [72]. Their ease of administration even enabled their use in toddlers and neonates [73], while their number and width allowed for precise control over dosage and release characteristics. All these favourable characteristics led to a high interest in producing 3D printed mini-tablets, loaded with various APIs, predominantly cylindrical shaped, and with diameters starting from 1.5 mm up to 4 mm [41,42]. In another study, hexagon-shaped tablets were gradually reduced in size, from 12 mm to 4 mm diameter [52]. Mini-caplets were also reported, devised by researchers as age-appropriate formulations, to replace the conventional capsules which lack dose flexibility [50]. While the compressed mini-tablets are considered up to diameters of 3 mm [74], some authors referred to their products as mini-tablets or mini-caplets even at higher diameters of up to 10 mm [40,50]. Given their small sizes, a limitation of these dosage forms would be the inability to accommodate high doses of API. Because of the low unit doses, conventional compression preparation faces difficulties in ensuring the homogeneous compression of mixtures and content uniformity [75]. In contrast, FDM 3D showed the ability to create mini-dosage forms with distinct drug doses of 2, 5, and 8 mg, consistent in mass, content, and API release rate [49] and proved the ability to titrate doses in increments as small as 0.19 mg through single-unit minitablets [41]. The size is important for ease of administration reasons; however, it can be connected to process-related issues, for example, the printed shape was found to be more irregular with the decrease in diameter [42]. Additionally, as a function of the dosage form and composition, size can have a variable impact on drug release. For example, in comparison to the lower dimensions (7.5 mm and 5 mm), the mini caplets printed with the maximum dimension (10 mm) demonstrated a more prolonged release of API over time, making them perfect for a modified-release dosage form [50].

As orodispersible dosage forms can increase the bioavailability of a drug, and prescribing them can improve patient adherence and compliance, they are becoming increasingly common in the pharmaceutical industry [76]. Owing to their quick disintegration in the mouth, orodispersible dosage forms meet the demands of paediatric patients who have swallowing issues [1]. Rectangle-shaped orodispersible films were prepared by FDM and compared to those obtained by other methods: casting and electrospinning. The 3D printed films demonstrated better mechanical properties, which improved after storage. Although there was no sign of API recrystallization in the 3D printed products, the released API recorded a two-fold decrease after storage for a month [53].

Cylinder-shaped and paediatric-friendly-shaped (smurf, palm tree, cherry, and banana) prints were developed as chewable formulations, with sizes of about 10 to 20 mm and a 12.5 mg dose of the API. The child-friendly designs, due to their irregular shapes, can also impact product properties. Tabriz et al. demonstrated that “smurf” designed tablets had the quickest API release, which was followed by the banana, cherry, palm tree, and then the regular design [43].

For the development of all paediatric dosage forms, but especially for orodispersible and chewable formulations, taste must be considered as an important quality attribute. The taste of medicines is a very important factor in children’s acceptability, which is why the exploration of taste-masking methodologies for compounds characterised by an inherent bitterness is necessary in this domain. A lot of APIs have a strong taste, which makes them unpleasant for children [77]. For instance, the exceedingly bitter diphenhydramine hydrochloride (DPH) was associated with HPC and palatability increasing excipients like sucralose as a sweetener and strawberry flavour. None of the subjects of the taste evaluation panel reported any disagreeable taste, and the printed products provided great taste masking. While most of the subjects gave positive scores for aftertaste, the examination of the 3D fruit chews revealed a sweet, fruity intensity. The findings could be explained by the sweetener and strawberry flavour’s synergistic interactions, which resulted in a mixture with a higher overall intensity than either of its constituent parts [43].

Another taste-masking strategy was the use of Eudragit E PO at ratios between 5 and 20%, known for its functions in paediatric dosage forms, among which are an increase in swallowability and masking of unpleasant odours [67]. In contact with the oral mucosa, the API had to diffuse throughout the polymers, thus its taste and smell was effectively masked. According to Fourier Transform Infrared Spectroscopy (FTIR) analysis, the physical trapping of the API in the polymer matrix after HME may also hide the strong taste [47].

Non-oral dosage forms such as suppositories for children were also prepared through FDM. For the paediatric population, the rectal route can be a useful substitute for the oral route because these dosage forms do not require taste masking or swallowing. Rectal forms can also be given to children who are unconscious, vomiting, or in an emergency [78]. Extrusion and printing were carried out below temperatures at which thermal events occur to avoid the amorphization and recrystallisation of the components, which might cause stability difficulties. Suppositories had the conventional torpedo design adapted to mimic infant-sized alveolae. One study reported their preparation from an API-loaded filament [45]. Another study reported the 3D printing of placebo PVA shells followed by the injection-filling of API-PEG mixtures [56]. It was found that changing the suppository’s dimension or infill density to vary the dose of prednisolone between 6 and 30 mg had no discernible impact on the release profiles [45]. The key aspects of the aforementioned studies are summarised in Table 2.

## 4. Principles and Advances in DPE

### 4.1. Technology

DPE is a material extrusion 3DP process which has been introduced as a potential replacement for FDM printing in the plastics industry. To print directly without first preparing filaments, this method entails extruding pellets or powders that have previously been melted in a “melt generation unit” through the die of a single-screw extruder to the printer’s nozzle. In this manner, the amount of equipment required to process the formulations is decreased. Furthermore, this method allows the extrusion of mixtures that FDM is unable to print because of the improper mechanical properties of the created filaments. When compared to FDM, the thermal stress that DPE-processed APIs undergo is noticeably lower [79]. One of the many applications of DPE 3D printing is the preparation of oral dosage forms; in this case, the target population for the following studies was paediatric patients, and the focus was on diseases which affect children, for which child-targeted medicines are scarce or nonexistent.

### 4.2. APIs and Doses

Various APIs were processed by DPE, either individually, such as budesonide [80], praziquantel [81], clobetasol propionate [82], ibuprofen [83], biotin [84], and captopril [85], or in combinations, such as ritonavir with lopinavir [86]. In these studies, researchers aimed for dose customisation [80] or for taste-masking [81]. Most of the research did not precisely specify the dosing range but included the percentage of API loaded into the dosage forms. The smallest recorded dose was 125 µg [82], while the highest was 100 mg [81].

### 4.3. Excipients

As polymeric carrier materials, HP-β-CD [82], Kollidon VA 64 [81], PEO 100000, HPC (Klucel ELF) [84], and PVA with HPMC [85] were mentioned, and each demonstrated an appropriate performance in DPE. A study by Tabriz et al. evaluated the taste-masking properties of a series of polymers: Soluplus^®^, PVP VA-64 and Eudragit EPO. All of these showed great taste-masking capacity of the bitter API, ibuprofen [83]. A critical parameter that conditions easy processing through DPE is having a suitable powder flow. This was ensured by adding surfactants such as Span^™^ 20, Kolliphor SLS [81], magnesium stearate [86], or PEO [82].

The printing often resulted in solid amorphous dispersions with zero-order release kinetics. When DPE was compared to FDM 3D printing with lopinavir or ritonavir as model drugs, FDM led to considerable drug degradation (>30%) at 120 °C, the temperature needed to produce printable filaments. Because of this, DPE, which involved a decreased temperature of 80 °C, was chosen as the more appropriate technique. Superior dissolution properties were obtained compared to a commercial product, Kaletra. In the DPE 3D-printed dosage forms, the APIs persisted in the dissolved form, while for Kaletra, the API precipitated in the intestine. This demonstrated the advantage of 3D printing for these specific APIs [86]. Other researchers tackled the colon delivery of budosenide by printing a mixture of API, HP-β-CD, and PEG6000 coated with Eudragit FS 30D. Dose customisation was successfully achieved [80].

### 4.4. Dosage Forms and Acceptability

The DPE 3D-printed paediatric dosage forms presented in the aforementioned studies were varied, from spherical mini-tablets with a diameter of 6 mm [86], cylindrical mini-tablets with a 5 mm diameter [81], and capsule-shaped tablets [84], to mucoadhesive films of 20 mm diameter [82]. Compared to conventional pharmaceutical formulations for oral administration, buccal drug delivery has some unique properties: substantially higher bioavailability, a quicker rate of drug absorption, and greater compliance for patients with special needs [87]. When inserted in the oral cavity, they dissolve or adhere to the buccal mucosa to release the drug [88].

**Figure 3:**
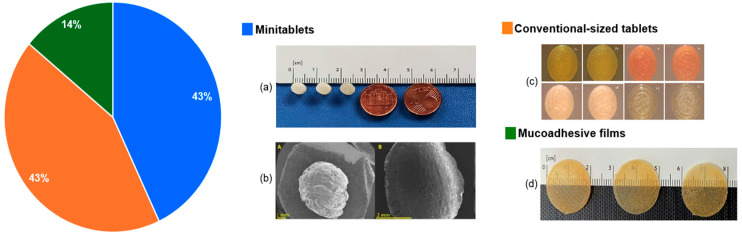
The distribution of papers reporting paediatric medicines manufactured by DPE 3D printing expressed as percentages as a function of dosage forms: minitablets (**a**) [80], (**b**) [86], conventional-sized tablets (**c**) [81], and mucoadhesive films (**d**) [82].

A conclusion of some of these studies was that, for most of the DPE formulations, good stability was reported for several months [81,82], with no drug degradation [80]. A pie chart with illustrations of the 3D printed dosage forms and the percentages of studies focused on these various dosage forms is presented in Figure 3, while a summary of these studies is offered in Table 3.

## 5. Principles and Advances in SSE

### 5.1. Technology

The SSE technique is appropriate for APIs that are susceptible to changes in temperature, as it eliminates the melting stage, thus reducing the likelihood of thermal degradation [89]. Although this is an advantage compared to FDM 3D printing, it is important that the heating temperature does not reach a high value to prevent excessive material softening and loss of shape during deposition [90]. Additionally, a range of pharmaceutical gel formers are available for the formation of the starting material. Semi-solid formulations can be readily prepared at pharmacies or hospitals, which makes this technique particularly helpful as a decentralised manufacturing method. Since solvents are the foundation of the printing formulations, a drying step is required to produce a solid product [89]. As a type of material extrusion 3D printing process, SSE produces objects of any size and shape by progressively depositing layers of gel or paste. A nozzle located at the syringe’s base is used to extrude the gel or paste during the printing process. One parameter that significantly influences printing accuracy is the nozzle diameter. Typically, one ought to select the smallest nozzle tip possible to facilitate simple material extrusion and create an item with a uniform surface. In addition to that, disposable syringes can be used to meet important quality standards for pharmaceutical use [91].

**Figure 4:**
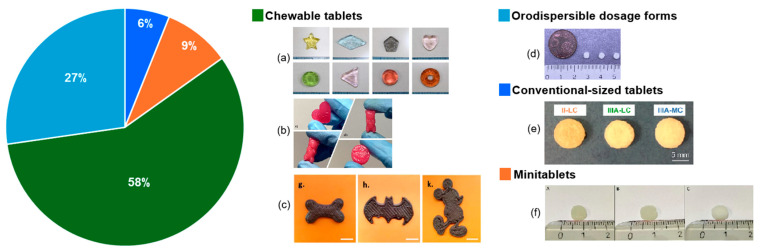
The distribution of papers reporting paediatric medicines manufactured by SSE expressed as percentages as a function of dosage form: chewable tablets (**a**) [95], (**b**) [102], (**c**) [104]; orodispersible dosage forms tablets (**d**) [109]; conventional-sized tablets (**e**) [92]; and minitablets (**f**) [93].

Several studies have focused on SSE 3D printing as a feasible technique for creating paediatric dosage forms. These studies are summarised in Table 4, and illustrations of some of the dosage forms can be seen in Figure 4. Paediatric patients’ compliance is significantly improved by SSE technology, because the semisolid material is ideal for making chewable tablets. Other than chewable dosage forms, mini-tablets and orodispersible dosage forms were also the centrepiece of many studies.

### 5.2. APIs and Doses

As specified before, the chosen drugs were selected based on paediatric requirements, either because there are no commercial products specifically for children, or because the doses for the commercial products are not proper and manual subdivision is required. SSE 3D printing studies comprised the following APIs: fenofibrate [92], sildenafil, furosemide [93], levothyroxine sodium [94], lamotrigine [95], amlodipine besylate [96], propranolol hydrochloride [97,98,99,100], spironolactone, prednisolone [99], ondansetron [101], ranitidine hydrochloride [102], isoleucine [103], ibuprofen [104,105], paracetamol [104,105,106], omeprazole [107], hydrochlorothiazide [108,109], levetiracetam [89], carbamazepine [110], warfarin [111], clopidogrel bisulphate [112], hydrocortisone [113,114], metformin [115], chlorpromazine hydrochloride [116], amoxicillin [117], isoniazid [118], dexamethasone [119,120], and a combination of sulfamethoxazole and trimethoprim [121]. Dosage forms with amino acids such as citrulline, isoleucine, valine, and an isoleucine and valine combination were also prepared specifically for metabolic disorders in children [122]. The doses ranged from a minimum of 0.46 mg/unit [110] to a maximum of 250 mg/unit [115] which demonstrates SSE’s advantage in flexibility compared to other 3D printing methods such as FDM. In that case, as specified in the HME and FDM chapter, the resulting mechanical properties of the filaments limit the drug load, since higher temperatures are used for a longer period. Furthermore, a study found that APIs with limited therapeutic indexes, such as warfarin, can be processed through SSE because of a low standard variation in drug content [111]. Another testament to the superior dose flexibility of SSE is that, when compared to manually subdivided dosage forms, 3D-printed medications with subdivisions from 1/10 to 2/5 led to a higher dose accuracy and minimal weight loss [94]. However, for some poorly water-soluble APIs such as fenofibrate, the final drug loads in the SSE 3D-printed tablets were relatively low, not as high as the desired doses for children. The researchers concluded that this 3D printing method is appropriate only for lipophilic medicinal molecules that can be administered at lower doses [92]. Some APIs face stability challenges which can be mended by using SSE. Omeprazole, for example, possesses a chemical instability; in an acidic environment it breaks down quickly, while in an alkaline environment it is stable [123]. By 3D printing gastro-resistant dosage forms incorporating pellets, omeprazole passes through the gastric acidic medium without degrading and arrives at the local point of action, the intestine, where it is absorbed into systemic circulation [107]. Another benefit for the APIs is dose-frequency reduction, which was demonstrated by creating dosage forms with a dual drug loading of ibuprofen and paracetamol. Both APIs were released at a comparable rate even though it is known that paracetamol dissolves faster in the stomach than ibuprofen [105].

### 5.3. Excipients

The proper excipients for this technique differ from the ones presented in the first chapters because here, a semisolid gel or paste needs to be prepared before extrusion, and for this, some solvents or hydrophilic excipients are necessary. First of all, hydrophilic excipients such as HPMC [94,95,120], gelatine [95,97,101,102,105,107,117,119,121], glycerine [96,101,105,107,117], carrageenan [102,105,107,118,119], pectin [101,122], pullulan [116], xanthan gum [102,107], and locust bean gum [105] were used as gelling or thickening agents to produce a stable gel or paste with sufficient viscosity. Curablend^®^ gel tablet base, a gelatine-based excipient, was also utilised as a semi-solid mixture-forming excipient [99,100,112,114]. In one study, three types of Curablend excipient bases were used, the aforementioned one as well as CuraBlend Water-Free Tablet/Troche Base and CuraBlend ODF Base [114]. Pregelatinized starch was another component of chewable tablets [121]. According to a study, gelatine was crucial for the structure and printability of the dosage forms. It also improved the chewable tablets’ masticatory qualities by decreasing adhesiveness and increasing cohesiveness and springiness. Carrageenan sped up preparation breakdown and increased the thermal stability of the formulation [98]. The delayed disintegration of locust bean gum, a galactomannan that functions as a gel-forming excipient in the embedded phase of SSE-produced chewable dosage forms was considered responsible for the similar release profile of ibuprofen and paracetamol [105]. Liang et al. found that the printability and thermal stability of the gel ink were enhanced by combining sodium carboxymethyl starch (CMS-Na) and carrageenan, which improved the gel ink’s viscosity and thixotropy [97]. Other excipients such as lactose monohydrate and PVP K30 were used as binders or thickeners in various studies [101,108,109,110]. Furthermore, a hydrophilic matrix with quick release properties, polyvinyl alcohol-polyethylene glycol graft copolymer (PVA-PEG, Kollicoat IR) was chosen by Aita et al. as the main excipient [89]. For lipophilic APIs, emulsion-forming lipid-based excipients were selected as the most appropriate, and those most frequently cited were Gelucire 48/16, Gelucire 44/14, Maisine^®^ CC, Captex^®^ 355 EP/NF and Capmul^®^ MCM EP [92,93,113]. These excipients are known to enhance the oral bioavailability of poorly water-soluble drugs [124,125,126,127]. Johannesson et al. demonstrated that the favourable characteristics of lipid-based formulations were preserved in emulsion gel-based 3D-printed products [92]. Chocolate is appealing to young patients, hence chocolate-based chewable dosage forms were developed via SSE, instead of using conventional excipients. Corn syrup was utilised to enable the 3D printing process of the chocolate-based dosage forms [104]. In addition to these main excipients, disintegrants such as croscarmellose sodium, Ac-Di-Sol^®^, and sodium starch glycolate (SSG) with a minimum concentration of 5% and a maximum concentration of 40% were added in the formulations [108,109,110]. As solubility enhancers, Kolliphor EL and Tween 85 were chosen as the most appropriate [92], and the only plasticisers used in these studies, as they are not considered a necessary excipient for SSE, were polysorbate 80 [93,114] and PEG [120]. Another particularity of paediatric dosage forms is the need for an appealing taste and taste-masking of bitter APIs. Sweeteners such as reduced syrup [95], glucose [116], sucralose [96,121], corn syrup [104], mannitol [116,121]; or as a diluent [120], sucrose [110,116,122], maltose [116], maltodextrin [122], isomalt [116], xylitol [118], maltitol syrup 80/55 [118,121], acacia honey [117], saccharin, sorbitol [119], and flavouring agents were typically used in low concentrations for this purpose [96,97,101,102,103,107,108,109,119,121]. In addition to that, Precirol^®^ ATO 5 was also chosen as a taste-masking agent in a study which produced mini-tablets with hydrocortisone, a bitter API [113]. Gamma-aminobutyric acid (GABA) was tested as a taste-masker for another bitter API, dexamethasone, and demonstrated its efficacy [119].

### 5.4. Dosage Forms and Acceptability

The produced oral dosage forms included conventional-sized tablets [92,112], mini-tablets [93,94], chewable dosage forms [95,96,97,99,100,101,102,103,104,105,106,107,114,117,118,119,121], troches [114], and orodispersible dosage forms [89,108,109,110,111,114,120]. Chewable oral products are a large class of dosage forms applied primarily to the oral cavity, with the mucosal lining of the oral cavity serving as the major site of absorption [128]. These dosage forms were a subject of interest for many researchers. Chewable dosage forms were 3D printed to respond to poor paediatric medication compliance and the weak swallowing abilities of the targeted population. They aimed at improving the sensory quality of the products and at reducing the number of excipients, to enhance the safety of the formulations. Various designs were proposed, from the regular ones, such as cylindrical-shaped [103,114,119,121], or square [106], or even more appealing options shaped like cartoons [104], bears [96,97,102,117], stars [95,104], hearts [95,96,97,102,107], diamonds [97], flowers [96,97], or depicting food, for instance, a lemon slice [107] and a donut [95]. The sizes ranged between 0.4 mm [96], designed for small children, and about a 20 mm diameter [97,102,105,106]. As for colour and flavour, a study by Goyanes et al. described six different kinds of dosage forms with various flavours and colours: strawberry, red; lemon, yellow; orange, orange; raspberry, light blue; banana, light green; and coconut, black. It was shown that the coconut flavour was not well-liked by patients, which may stem from its lack of popularity in the region of study [103]. Because chocolate is known to appeal to children, some researchers focused on preparing brown chocolate 3D-printed dosage forms. A study compared molded dosage forms to 3D printed ones and concluded that, in line with the Food and Drug Administration’s (FDA) Chewing Difficulty Index (CDI) values stated for commercial chewable tablets, both versions of the dosage forms had a CDI that varied from 0.15 to 0.28 nm, but only the 3D-printed tablets passed the rest of the quality tests [106]. Through embedded 3D printing, bespoke chewable dosage forms were created by Rycerz et al. with an appealing Lego-like brick design. An embedded phase consisting of the model drug’s ink was extruded into a gelatine-based matrix [105]. Researchers also focused on producing another category of 3D-printed dosage forms: orodispersible formulations. SSE 3D printing of orodispersable solid formulations was a subject of interest for a series of studies, because these dosage forms improve swallowability through disintegration in the oral cavity and can have customised doses, which are necessary features for children. The paediatric orodispersible tablets had cylindrical [89,108,109] or round [110] designs with a diameter of less than 10 mm, while the films printed through SSE were round and had a maximum volume of 200 mm^3^ [111]. A limitation of one of the previously mentioned investigations was the tablet size. Tablets with a 10 mm diameter are not appropriate for use with younger paediatric categories, such as neonates and infants. To make administration in the paediatric population possible, studies proposed increasing the amount of API in the formulation or dividing the dose into numerous units [89].

**Table 4:** SSE 3D-printed paediatric dosage forms.

API Name, %, Dose/Unit	Excipients		Design	Size/Volume	Observations	Ref.
	Semisolid-Forming Excipients	Others				
**Tablets**						
Fenofibrate, 41.9/45/53.2 mg/unit	Maisine CC/Captex 355 EP/NF/Capmul MCM EP/Soybean oil 32.5%	Croscarmellose sodium 5%—disintegrant Kolliphor EL, Tween 85 35%—solubilizers	Cylindrical	7.9 or 8.5 mm diameter	- The lipid-based formulations’ redispersion was successful - Low drug loads restricted the possibility of printing tablets with a high API dose	[92]
1% or 2%, Clopidogrel bisulphate, 2–10 mg/unit	Curablend 94.6%/94.8%	Polysorbate 80 2%—plasticiser Citric acid 1.2–2.4%—pH regulator	Round	Various sizes, n.s.	- Tablets had rapid drug release - disintegration was under 10 min - The clopidogrel–excipient mixture demonstrated chemical stability	[112]
**Mini-tablets**						
Sildenafil, 4 mg/unit or Furosemide, 2 or 10 mg/unit	Gelucire 48/16	Polysorbate 80—plasticiser	Cylindrical	5 mm diameter	- The quality requirements of the European Pharmacopoeia were assessed, and it was decided that the uniformity of mass can guarantee the mini-tablets’ quality	[93]
Levothyroxine sodium, 0.061%	SH-E30 (HPMC) 5%		Round	4.8 to 7 mm diameter	- SSE 3D printing was evaluated as a possible replacement for manual subdivision, and it was deemed appropriate with a maximum weight loss of 3% and a high dose accuracy - 3D printed tablets were found to be stable for at least three months	[94]
Hydrocortisone	Gelucire 44/14, Precirol ATO 5, 70%/30%, 60%/40% or 50%/50%		Cylindrical	1.5 × 6 mm	- Micro-extrusion was carried out, with a nozzle of 1.5 mm - Precirol ATO 5 had a double role: semi-solid forming excipient and taste-masking agent - The API dissolving rates were adjusted by varying the ratio of the two excipients, resulting in sustained release mini-tablets	[113]
**Chewable dosage forms**						
Lamotrigine, 10–20 mg, <1 mg/unit	HPMC 100/200/300 mg Gelatine 0.5/0.75/1/1.5%	Reduced syrup 4.29 g—sweetener Water 8.71 g	Star, trapezoidal, triangular, circular, donut, heart, etc.	About 10 mm	- For the formulations with low HPMC or gelatine content, the viscosity was reduced, and printing was challenging	[95]
Amlodipine besylate, 1.5–5 mg/unit	Glycerine 10%	SSG 7%—solubility enhancer CMC Na 1%—thickening agent Sucralose 0.1%—sweetener Lemon essence 0.05%—flavouring agent	Cartoons (flower, heart, bear, etc.)	0.4 to 0.6 mm diameter	- Taste-masking was efficient - The dosage forms were found to be stable for at least four months	[96]
Propranolol hydrochloride, 0.455%, 1–5 mg/unit	Gelatine 12% Carrageenan 0.65%	CMS Na 6% Flavouring, colourants	Capsule, diamond, flower, bear	20.3 mm diameter	- CMS-Na and carrageenan improved the gel ink’s viscosity and thixotropy - Immediate release was achieved - Taste-masking was accomplished	[97]
Propranolol, 1%/Spironolactone, 1%/ Prednisolone, 1%	CuraBlend		Cylindrical	Less than 20 mm diameter	- Uniformity of 500 mg showed less mass variation than lower weights, but all of the tablets complied with Ph. Eur. standards - Over the course of nine months, stability showed almost no fluctuation - Prednisolone tablets had the slowest rate of drug release, followed by propranolol and spironolactone - Transferring the liquid form of these tablets through a nasogastric tube was successful	[99]
Propranolol, 1%, 3 mg, 4 mg, or 5 mg/unit	CuraBlend		Round or oval-shaped	Less than 20 mm diameter	- CMS-Na and carrageenan improved the gel ink’s viscosity and thixotropy - Immediate release was achieved - Taste-masking was accomplished - Adding the API to the semi-solid CuraBlend mix reduced viscosity and boosted fluidity - The SSE technique was automated, and three specific tablet weights were targeted	[100]
Ranitidine hydrochloride, 1.004 g, 28 or 32 mg/unit	Xanthan gum 0.075 g Gelatine 2.4 g Carrageenan 0.6 g	Corn starch 0–1.5 g Strawberry essence 0.15 g Sweetener 1 g Purified water	Heart, bear, disc	15 to 20.8 mm diameter	- Starch-free formulations produced a fast release of ranitidine, whereas integrating corn starch produced a slower release	[102]
Isoleucine, 14.4%, 50 to 200 mg/unit	Pectin Maltodextrin	Flavouring agents Colourants Water	Cylindrical	10 mm diameter and smaller	- Children’s preference was assessed, and the most-liked formulation was the orange colour and taste - Clinical study in which the isoleucine mean blood levels were within the desired range - Tablets were stable for at least one month	[103]
Ibuprofen, 19.6 mg/g or Paracetamol, 22.9 mg/g	Chocolate, Corn syrup (1:1 *w*/*w*)	-	Star, cartoon characters		- Immediate release profile for both APIs (hydrophilic paracetamol and lipophilic ibuprofen)	[104]
Paracetamol	Chocolate	-	Square	8 × 8 × 5 mm to 22 × 22 × 17 mm	- 3D printed tablets were compared to mold-cast dosage forms - In line with FDA’s CDI values stated for commercial chewable tablets, both versions of dosage forms had a CDI that varied from 0.15 to 0.28 nm - 3D printed tablets passed all the quality requirements evaluations and allowed dose flexibility	[106]
F1: Omeprazole,1%, 7 mg/unit F2: Omeprazole, 22.5% pellets, 11 mg/unit	F1&F2: Carrageenan 2%, Xanthan gum 0.5%, F1: Glycerol 15% F2: Gelatine 8%	F1&F2: Sweetener, essence, lemon juice, water F1: CMC 3%—thickening agent, sodium bicarbonate 2.5%—to adjust the pH	Disc, lemon slice, heart	-	- The SSE 3D printing technique and the fluid bed pellet coating were combined - 3D printed hydrogels loaded with gastro-resistant omeprazole pellets (F2) were compared to hydrogels containing dissolved omeprazole (F1) - By using pellets, the bad taste of the API was masked - Only F2 was found to be gastro-resistant	[107]
Ibuprofen, 28%, 12–76 mg/unit Paracetamol, 40%, 6–77 mg/unit	Embedding medium: Glycerol EP: Gelatine (25:30) and water Paste: 2.98% Locust bean gum solution	Food dye	Lego-like brick	20 mm diameter	- In this study both drugs had a similar release, although paracetamol is generally known to dissolve faster, the reason would be the delayed disintegration of the locust bean gum solution - Sweet flavour of the printed dosage form was identified	[105]
Metformin, 250 mg/unit	Gelatine 20%	Starch 5%	Rectangular	23.8 mm/24.5 mm diameter, 4.3 mm/6.1 mm height	- A visually pleasing design was achieved - A rapid onset of the API’s action was observed, which can have an advantageous control for post-prandial glycemia in children compared to conventional dosage forms - In vivo studies should be further conducted to demonstrate the printed dosage form’s applicability	[115]
Amoxicillin, 200 mg/unit	Corn starch and glycerol	Sweetener, plasticiser and flavouring agent: acacia honey, 30%	Bear-shaped	12.90 × 16.94 × 5.44 mm (width × length × height) after drying	- Use of naturally derived components with the safety of the formulation for children in mind - A sensory analysis of four different types of placebo gels was conducted, and most of the participants chose the formulation with the highest amount of honey (30%) - PermeaPad^®^ barrier was used as a model membrane for the prediction of API oral absorption	[117]
Isoniazid, 5%	Gelatine, carrageenan gum	Sweeteners: maltitol and xylitol, flavouring	Cylindrical	5.6 mm height and ~12 mm diameter	- Low printing speeds resulted in warping - An evaluation of taste-masking with an electronic tongue was conducted and the findings denoted a balanced taste of the printed dosage forms	[118]
Sulfamethoxazole 13.7% single-layer and 27.4 bilayer, Trimethoprim 2.7% single-layer and 5.4% bilayer, 100/20 mg single layer and 200/40 mg bilayer	Gelatine and pregelatinized starch	Water, osmotic filler: mannitol, sweeteners: sucralose, maltitol syrup 80/55, colouring and flavouring agent	Cylindrical	14 × 8 × 5 mm (length × width × height)	- Single-layer tablets yielded a potential bioequivalence to commercial products with the same APIs - Bilayer technique is promising for taste-masking the bitter API; however, the printing process needs refining, and the long-term stability has to be assessed in the future - The formulation is clinically feasible due to its enhanced palatability when compared to conventional oral suspensions, demonstrated by patient adherence testing	[121]
Ondansetron, 4 mg/unit	Pectin, gelatine	Thickener: PVP, plasticiser: glycerol, pH modifier: citric acid, conserving agent: potassium sorbate, sweeteners, flavouring, colouring	Cylindrical	10 mm diameter and 4.5 mm height	- It was demonstrated that the ondansetron content and model size had a linear relationship	[101]
Dexamethasone, 2 mg and 12 mg/unit	Gelatine, carrageenan, Xanthan gum Pectin	Water, sweeteners: sorbitol solution and saccharin sodium, bitter-blocker: GABA, citric acid, polysorbate 80, raspberry flavouring, colourant Sweeteners: sucrose, maltose, maltodextrin, colourants, various flavours	Cylindrical	Varying diameters and heights	- The tablets were specifically designed for nausea in paediatric oncology patients - GABA was an effective taste-masker in combination with this API	[119]
Amino acids: Isoleucine 20%, 22.5%, 40%; Valine 17.5%, 20%, 40%; Citrulline 30%, various doses			Cylindrical	~1 cm to 6 mm diameter	- A clinical study was conducted - The chewable dosage forms’ plasma levels of amino acids were akin to conventional formulations’ levels - Shape and texture of chewable dosage forms were well-liked by patients	[122]
**Orodispersible dosage forms**						
Hydrochlorothiazide, 40.40%, 10 mg/unit	PVP 8.10% Lactose monohydrate 18.20%—binders	Ac-Di-Sol 30.30%—disintegrant Banana flavouring essence 3%	Cylindrical tablets	5 mm diameter	- Molding and SSE 3DP were compared - All 3D printed dosage forms complied with the Ph. Eur. standards - DSC demonstrated that 3D printing resulted in a more compact and stable structure	[108]
Hydrochlorothiazide, 40.40%, 10.32 mg/unit	PVP K30 8.1% Lactose monohydrate 18.2%—binders	Ac-Di-Sol 30.30%—disintegrant Banana flavouring essence 3%	Cylindrical tablets	About 5 mm diameter	- Different printing surfaces were tested (steel, glass, polypropylene, blue tape, and methacrylate) out of which polypropylene and glass were the most fitting - The disintegration time was modified by changing the infill percentage - The weight of the dosage forms was constant	[109]
Levetiracetam, 27.6 g	Water PVA-PEG (Kollicoat IR) 31 g-hydrophyllic matrix	-	Cylindrical tablets	10 mm diameter	- A limitation of this study was the size of the tablets, which is not appropriate for younger paediatric patients (neonates, infants)	[89]
Carbamazepine, 0.46 mg/unit	Water Lactose monohydrate 50%—diluent Kollidon VA 30 5%—binder	SSG 40%—superdisintegrant Sucrose 5%—sweetener	Round tablets	3 mm diameter	- The taste of the printed dosage forms was assessed, and it was determined that the bitter taste of the API is indistinguishable - Advantages such as rapid breakdown and fractioned dose are attributed to the resulting dosage forms	[110]
Clorpromazine hydrochloride, 2.5%	Pullulan 48.8–50%	Sucrose, maltose, isomalt, glucose, fructose 48.8–50%	Round films	20 mm × 30 mm × 0.8 mm	- Oromucosal films were successfully developed - Five sweeteners were evaluated by a human taste panel, and sucralose was the favourite - E-tongue sensor test results were in agreement with those delivered by human panellists	[116]
Warfarin, 3.9 to 7.4 mg/unit	PVA or HPC 20%		Round films	25–200 mm^3^	- The PVA films were extremely curved and stiff after drying, making them unsuitable for use as orodispersible films - The neutral surface pH of both the drug-loaded and unloaded films suggests that they can be used inside the oral cavity without causing discomfort	[111]
Dexamethasone, 1%/3%, 0.25 to 5 mg/unit	HPMCAS	Diluent: D-Mannitol, D-Sorbitol, plasticizer and suspension stabiliser: PEG, superdisintegrant: CMC, flavour enhancers: citric acid and rebaudioside A	Cylindrical	Various sizes	- GRAS excipients were exclusively used - Ink homogeneity was determined by taking multiple samples from different places in the syringe	[120]

HPMC, hydroxypropylmethylcellulose; SSG, sodium starch glycolate; CMC, carboxymethylcellulose; PVP, polyvinylpyrrolidone; PEG, polyethylene glycol; HPC, hydroxypropylcellulose.

## 6. Other 3D Printing Techniques

Dosage forms used in the paediatric pharmaceutical domain were also 3D printed via some less utilised, but highly practical methods. These include drop-on-demand (DoD)/drop-on-powder 3D printing, also known as binder-jet 3D printing, a sub-technique of inkjet printing, selective laser sintering (SLS), and 3D bioprinting.

When utilising inkjet printing, layers are created by spraying a liquid binding agent onto a bed of powder material. This paste-like liquid binding agent is applied as droplets over a powder bed with exact movements, speeds, and sizes. The sprayed liquid is made up of excipients, solvents, and/or APIs. There are two types of inkjet printing: DoD and continuous inkjet (CIJ). Droplets ranging in diameter from 10 to 50 μm are produced using the DoD approach. Piezoelectric crystals are employed as printer heads [129]. The development of high-potency drug products, combination drugs containing several APIs, and pharmaceutical products catered to a particular type of patient can all benefit from the application of DoD technology, which provides accurate control of material characteristics, API solid state form, drop dimension, and dynamics [130]. A subtype of DoD, the drop-on-solid deposition process, also known as plaster printing, drop-on-powder, or binder jetting, entails depositing a binder droplet on the powders. A roller applies another layer of powder automatically when the previous layer is finished [129]. Spritam^®^, the first 3D printed medication approved by the FDA and introduced to the market in 2016, is a testament to the advancements made in inkjet printing [131]. Some researchers focused on employing this method to produce paediatric oral dosage forms. A study conducted by Sundarkumar et al., focused on developing small-size dosage forms with appealing qualities including flexible drug release and dosing and ease of swallowing, rendering them a great option for children. To create individual mini-tablets, a DoD system was used to make precise droplets with a melt-based formulation. These droplets were then caught and solidified in an inert solvent bath. The bath material used in the production process was silicon oil. Lisinopril and atorvastatin were selected for the purpose of this study. PEG was used as a binder and plasticizer, Gelucire as a surfactant, and Kollidon TPGS as a solubilizer. These were the excipients of choice because of their low melting points (between 40 and 70 °C), which guaranteed the solidifying of the dosage form at room temperature. Higher molecular weight (MW) PEGs functioned as binding agents in these 4 mm spherical mini-tablets, while lower MW PEGs functioned as disintegration agents to enable the fast release of the API. The production of large-dose tablets was the challenging part of the process. The printing of 10 mg doses was attempted in this investigation; however, formulations with drug strengths of more than 3 mg produced a very thick paste-like material that was unprintable. Conversely, doses of 0.1 mg were successfully printed. Finally, another important drawback was the low hardness [132]. Another study investigated the feasibility of developing a dynamic dose-control system for theophylline and metoprolol tartrate using a desktop 3D printer. The percentages of APIs used in their respective formulations were 35% theophylline and 10% metoprolol. Additionally, the effects of four dose-regulation techniques—dividing by 3D printing, splitting, powder subpackaging, and liquefaction—on the precision of drug dose and in vitro drug release behaviour were studied in this work. The size of the 3D-printed tablets varied from a few mm to more than 10 mm. The accuracy ranged from 91.2 to 108% when printing tablets with the desired drug doses (from 2 mg to 240 mg). It was discovered that the printing ink’s ideal concentration range was 2.0–10.0 mg/mL. The most important finding of the study was that the 3D-printed tablets had the highest dose accuracy and the drug content that was closest to the intended dose, irrespective of API, followed by the liquefaction process, which was deemed the next best approach [133]. A prototype modular system for producing customised solid oral medicinal formulations via drop-on-demand (DoD) printing was developed by researchers. The continuous and semi-batch modes of the system’s operation were demonstrated for the production of mini-tablets with PEG 2000 as the main excipient and atorvastatin as the API [134].

During long-term drug treatment, children with epilepsy may miss or take late doses of medication; therefore, it is important to promptly make up for these missed doses [135]. The optimal remedial dose based on the physiologically based pharmacokinetic (PBPK) model in children to ensure the stability of the blood concentration in the event of missing doses was investigated in the following study. Dogs were used in in vivo pharmacokinetic investigations using prefabricated, binder-jet 3D-printed (BJ-3DP) levetiracetam instant-dissolving tablets. The PBPK model was utilised to predict the dose of levetiracetam in children instead of the weight. After carrying out a bioequivalence study, it was found that the 3D-printed tablets were bioequivalent to commercially produced Spritam in both dogs and humans. Additionally, this study provided guidelines for the tailored treatment of paediatric epilepsy patients [136].

Customised acetaminophen orally disintegrating tablets with intricate architecture were 3D printed with specific populations in mind, including children. For this reason, the surface of the dosage forms was embedded with braille for visually impaired children, and with cartoon figures, text, and QR codes. The inner core had a loose powder structure, and, for paediatric patients, the dose of the tablets was visible on the back, while on the front, cartoons or animals were depicted to improve acceptability [137].

Because of BJ-3DP’s limitations, including insufficient powder feeding and scraping, lack of appropriate ink, nozzle blockage, binder migration, and bleeding, another similar technique was investigated [138]. Children’s colourful cartoon tablets with levetiracetam were created using a novel colour jet 3D printing (CJ-3DP) technique, and the likelihood of scale-up was assessed as well. Generally, when using multiple printing heads equipped with different printing inks, CJ-3DP can achieve a more refined design for preparation compared to BJ-3DP. In this study’s process, microcrystalline cellulose served as a filler and disintegrant, mannitol was employed as a sweetener, and sucralose and spearmint flavour as flavouring agents. The glidant was silicone dioxide in colloidal form. A PVP K30 printing ink binder was utilised with glycerine as the plasticizer and polysorbate 20 as the wetting agent. Various concentrations of these excipients were tested, but the final printing ink composition was 40% (*v/v*) isopropanol aqueous solution with 4% (*w/w*) glycerine and 0.05% (*w/w*) PVP. The primary solvent was isopropanol. The API doses were 250 mg and 1000 mg. The outcomes demonstrated that, while PVP added to printing ink did not increase tablet hardness, it did substantially decrease tablet friability. Three different forms of internal spatial structure models—lattice structure, hollow structure, and hollow structure with inner support—were created to further expedite API release. The hollow structure was chosen. Finally, the cartoon tablets had different designs (rabbit, circular, heart, and bear), out of which the rabbit-shaped one was considered inappropriate for future studies because of its protruding parts—the ears–which can easily break [139].

Another 3D printing method used for developing paediatric dosage forms was inkjet bioprinting. This technique uses ejecting ink droplets to print. Alginate hydrogel is the material that is most commonly used [140]. Kondiah et al. designed and manufactured a novel drug delivery system for children. To create a stable, 3D-printable bio-ink, methotrexate-loaded PLGA nanoparticles functionalized with TPGS were embedded in a sodium alginate—gelatine hydrogel. After that, a 3D-printed tablet intended for oral chemotherapy applications was made using this mixture. According to the findings, the ink containing 10% gelatine and 8% sodium alginate provided the best printing accuracy. An improved bioavailability of methotrexate was achieved, because TPGS inhibited the P-glycoprotein and stabilised the molecule’s conformation. The concentration of the optimised formulation of methotrexate-loaded nanoparticles was 50 mg within 50 mL of hydrogel. The sodium alginate–gelatine nanoparticles in a buffer with a pH 6.8 showed patterns of sustained release, whereas the drug first displayed a burst release in the first sixty minutes at pH 1.2. This suggested that these dosage forms may offer promising gastrointestinal absorption of methotrexate [141].

Compared to other 3DP techniques, SLS technology has numerous benefits for printing pharmaceuticals. This solvent-free printing process can print at a reasonably high speed and does not require the use of filaments as raw materials, polymerizable liquid binders, or post-processing. Its solvent-free nature makes it perfect for including therapeutic compounds that are sensitive to organic solvents and water. Furthermore, as there are no post-processing steps involved other than harvesting printlets from the loose powder, printlets are immediately available for dispensing after printing. The method’s sole requirement is that the formulation’s components be thermoplastic and thermally stable. SLS uses infrared and laser energy. The three primary factors influencing the effectiveness and quality of the SLS printlets were chamber temperature, temperature of the surface, and laser velocity [142]. In fragile populations, SLS generally demonstrates a great deal of promise for increasing compliance [143]. One example in which SLS 3D printing could be used is in the treatment of Acquired Immune Deficiency Syndrome (AIDS). To stop AIDS symptoms, patients need to take antiretroviral drugs for the duration of their lives [144]. The secret to the effectiveness of AIDS therapy is, thus, adherence to the drug regimen. As few drugs benefit from easily available and flexible paediatric formulations, Kayalar et al. aimed to obtain paediatric-friendly, dose-flexible orodispersible drug delivery systems for tenofovir disoproxil fumarate to potentially be used in hospitals with other antiviral medications. The composition was made up of 16.3% API, 72.7% PVP K16-18, 8% magnesium aluminium silicate, 0.3% colloidal silicon dioxide, and 3% Candurin^®^ NXT Ruby Red, the melting component. It was shown that the poor spreadability caused by printing above a surface temperature of 85 °C led to uneven powder deposition and agglomeration. The selected laser speed range was 200–240 mm/s. Apparently, the density of the tablets decreased with increasing laser speed. Furthermore, the in vivo performance of the printlet was compared to the generic Viread^®^ tablets, and they could be considered bioequivalent [145]. Tenofovir disoproxil fumarate was combined with lamivudine in printlets created via SLS. A series of formulation and process factors were analysed, and it was concluded that this method has significant potential to enhance paediatric patients’ adherence [146]. Another antiretroviral drug, efavirenz, was the focus of a study developing dispersible 3D-printed dosage forms through SLS. These tablets are specifically designed to be delivered via the nasogastric administration route to children in need of nutritional support. Hydrophilic polymers Parteck MXP and Kollidon VA64 were selected as excipients, and the printlets had a final concentration of 20% and 40% efavirenz. Parteck MXP and Kollidon VA64 printlets exhibited distinct disintegration behaviours, demonstrating that each polymer characteristic can offer a unique approach to achieving a dispersible dosage form. It was discovered that Parteck MXP could be applied to rapidly disintegrating formulations [147]. An additional anti-HIV drug, lamivudine, was the focus of printlet development via SLS. Some process and formulation variables were tested, and the optimal printlets exhibited the same pharmacokinetic characteristics as commercial tablets, thus being considered bioequivalent. The dosage forms are expected to meet dissolving standards during the in-use period, and pharmacies may be permitted to dispense the medication for up to 90 days [148].

## 7. Practical Implications and Future Directions

Based on the information presented in the previous sections regarding the 3DP of paediatric medicines, it is reasonable to say that this domain has made huge steps in the last few years. However, significant progress is still needed to reach the full potential of this technology in the paediatric therapy field. While the high number of papers published in recent years confirms the existence of a dynamic, innovative research environment, input from pharmaceutical companies and regulatory authorities is needed to take the field forward and closer to the goal of promptly providing paediatric patients medication tailored to their needs. A comparative overview of FDM, DPE, and SSE technologies is presented in Table 5, summarizing key formulation and translational aspects relevant to paediatric drug development.

A few points arose from the performed bibliographic research:

- *Evolving 3D printing technologies*. As shown in the sections above, FDM, DPE, and SSE were the methods of choice for paediatric drug 3DP. Several review papers offer detailed descriptions of these technologies, with an overview of their background, process stages and equipment, process control, material-related information, and even an inventory of 3DP techniques tested on particular APIs [149]. However, their working principles and unique characteristics influence their suitability for prepare medicines for children. The table below gathers some important points to consider regarding the three technologies, which could guide the choice of a method when the preparation of 3DP paediatric drugs is considered.

Significant progress has been made in the 3D printing domain in recent years, and new technologies have emerged. For quicker implementation into clinical practice, new developments could focus on better control of the printing conditions and printing resolution and on ensuring the pharmaceutical quality of the printed products. Some research groups have already reported on results obtained with embedded near-infrared (NIR) spectrometers [154,155] or balances [84,156] to monitor the API content and API release and mass variation in the prints, respectively. One of these was a case study which demonstrated that, at the point-of-care, NIR can enable the safe, customised DPE 3DP manufacturing of medications by facilitating non-destructive, tablet-by-tablet quality control [154]. Moreover, a bilayer SSE technology was tested, showing promise in the taste-masking of bitter APIs and improving palatability, but, as of right now, the technology cannot guarantee an adequate printing process or scale up to clinical quantity levels. Future improvements are necessary to attain clinical scalability, as it is still an experimental but ingenious method [121].

- *improved excipient development and selection*. Excipients with specific properties are requested for each 3DP technology, and as discussed before, the performance of well-known conventional material is still to be understood in conjunction with different APIs and new 3DP technologies. However, the development of new excipients, tailored to undergo the stages of 3DP, with good safety profiles for all categories of paediatric patients, is crucial.

With respect to excipient selection, which is recognised as an important challenge in paediatric drug development, researchers and formulation pharmacists can use the safety information provided by the STEP database, which is continuously expanding [61,62]. Moreover, a systematic strategy centred on risk-management principles was recently proposed by a group of researchers under the name of the PERA tool to facilitate the choice of appropriate materials for paediatric preparations [157,158]. The lack of established frameworks for excipient selection frequently hinders the transfer of formulations from research to clinical use, making this a crucial, yet under-addressed aspect of clinical translation. Using systematic techniques, such as the PERA tool, could facilitate safer, more efficient paediatric 3DP formulations, expedite material selection, and support regulatory acceptance.

- *easy design and formulation*. Up to this point, formulation and design strategies have aimed at obtaining multiple dosage forms in various shapes and sizes with specific API release times to match the needs of children of different ages. The inclusion of nanosystems into the printed products has also been mentioned as a novel delivery strategy [141]. Future studies could tackle controlled release, varied nanosystem addition, and improvements in size, texture, palatability, and ease of administration. A thorough knowledge of children’s preferences and needs regarding taste, texture, colour, and administration skills would contribute to the development of 3DP drugs; therefore, more studies are required to collect patient feedback.

Specific software and online applications were released to ease the formulation of FDM printable mixtures containing one of the many tested APIs (more than 900), which brings the new technology closer to being able to be formulated by pharmacists with a limited background in 3DP [159]. In the future, computational models and simulation techniques that have already been used to predict and optimise drug release [159] could be extended to estimate and adjust texture, palatability, and drug adherence.

- *regulatory framework*. The approaches mentioned above cannot succeed without clear regulation concerning the 3DP drug production and clinical studies. The FDA initiated a discussion about point-of-care manufacturing. The published paper emphasises that point-of-care manufacturing requires solid quality systems and adaptable, risk-based Good Manufacturing Practice (GMP) frameworks designed for small-batch, bespoke production [160]. These ideas promote the safe, decentralised production of patient-specific medications with real-time quality monitoring using 3D printing of pharmaceuticals. EMA proposes recruiting specialised expertise, modernising regulations, tackling point-of-care manufacturing issues, and promoting flexibility in GMP application [161].
- *clinical translation.* Although FDM is the most studied academic approach with many formulation studies and geometry/acceptability trials, continuous in-line quality control for routine clinical use, commercialisation of standardised API-loaded filaments, and GMP-compliant implementations are still lacking. DPE is still limited by variable powder flow, non-uniform API distribution, and a lack of validated quality control methods. Prior to clinical implementation, feed systems, stability, and GMP/point-of-care conditions must be certified. Regarding SSE, some clinical studies were already performed on paediatric 3DP drugs developed and manufactured in hospital pharmacies with a GMP-certified 3D printer. They demonstrated, on the one hand, the need for this therapeutic alternative, but also the feasibility of point-of-care manufacturing of small batches of 3D-printed medicines and their acceptability [103,121,122]. Customised dosing was based on routinely monitored amino acid blood levels and guided the 3D-printed formulations. The focus was on comparing adherence and acceptability versus conventionally compounded medicines [122]. In addition to that, 3D printing is slowly but surely integrating itself as a safer and more effective way of preparing dosage forms when compared to compounding in community pharmacies. One study prepared capsules with minoxidil with a 3D printer by automatic filling, effectively reducing the time needed for production. The stability of the pharma-inks and capsules was also tested and found to be adequate [153]. This demonstrates SSE’s advanced technological maturity. Scaling-up, standardised regulatory procedures, and dependable automated QC are the remaining challenges, nonetheless.

## 8. Conclusions

This work shows how 3D printing technologies, especially FDM, DPE, and SSE, have made significant advancements towards creating specific dosage forms for paediatric patients. The role that these technologies play in meeting the special requirements of paediatric patients is highlighted, including increased drug adherence, better palatability, and accurate dosing. Paediatric medication administration problems can be effectively solved with the use of individualised mini-tablets, chewable forms, and orodispersible tablets or films for this kind of patient, who often has trouble swallowing, or a sensitive palate. Furthermore, the ability of 3D printing to modify drug release rates, size, shape, and taste-masking qualities offers an individualised approach to paediatric care that is lacking in conventional techniques. While promising, the study also shows that further investigation is required to completely include the use of 3D printing into paediatric pharmaceutical development, particularly in the topics of dosage forms’ long-term stability, excipient safety, and clinical effectiveness.

## Figures and Tables

**Table 1:** Shortcomings of medicinal products for paediatric use.

Route of Administration	Dosage Forms	Shortcoming	References
Oral	Solution and syrup	Excipient safety issues Taste, aftertaste, and smell issues Very small volumes used for younger children Measuring device needed Stability issues Misdosing issues when measuring the needed volumes	[20,27]
	Suspensions and emulsions	Excipient safety issues (solvents, sugar, flavours, dyes) Risk of medication errors because of the need to redisperse the API Thermodynamic instability	[28]
	Capsule	Swallowing issues because of their size Risk of choking Taste and aftertaste issues Dosing issues, opening is often needed	[27]
	Dispersible tablets, powders, granules, pellets or sprinkles for reconstitution	Instructions can be complicated when reconstituted in solvents Risk of local injury when the used volume of liquid is not appropriate	[24]
	Tablets	Swallowing issues because of their size Risk of choking Taste and aftertaste issues Dosing issues, splitting is often needed	[23]
	Chewable tablets	Dose flexibility needed Splitting is not possible Controlled release can be technologically challenging Taste masking is difficult Bioavailability may be changed by retention time in the mouth. Potential overdose if used improperly as a candy Patients should be instructed carefully when administering	[1,29]
	ODTs	Dose flexibility needed Splitting is prohibited because of their fragility	[1]
	ODFs	Mouthfeel and taste issues Controlled release can be technologically challenging Uniformity of dose can be difficult to achieve Only small doses can be incorporated	[1]
	Mucoadhesive films	Pharmacokinetic and pharmacodynamic challenges Standard- derived methods for determining in vitro, in vivo, and ex vivo mucoadhesive properties need to be determined before formulation	[30]
Nasal	Sprays, drops	Can cause damage of nasal mucosa The nasal cavity’s state impacts drug absorption	[31]
Ocular	Drops, ointments, gels, and inserts	Difficulty of administration Systemic side effect risk due to the fact that ocular dosing is not weight-adjustable Requirement for customised paediatric delivery systems to administer lower drug doses	[24,32]
Otic	Ear drops	Needs frequent use which can lead to poor patient compliance	[33]
Pulmonar	Aerosol devices	Devices intended for adults are adapted for children Very common off-label use Need for proper face-masks designed for children	[34]
Rectal	Suppositories	Portions of adult-use suppositories are used, leading to inaccurate doses, instability, improper shape for rectal insertion Undesirable route in children’s opinion	[24,35]
Injectable	Injectable solutions and suspensions	Excipient safety is imperative Higher costs Need for trained specialists for administration Acceptability challenges Serial dilution errors	[25]
Transdermal	Transdermal patches	Suitable only for some APIs Difficult to modify drug dose for premature neonates	[26]
	Transdermal microneedles	Complicated to design proper structures to target the various paediatric age groups	[36]

API, active pharmaceutical ingredient; ODT, orodispersible tablet; ODF, orodispersible film.

**Table 2:** FDM 3D-printed paediatric formulations.

API Name, Percentage, Dose/Unit	Excipients			Design	Size/Volume	Observations	Ref.
	Filament-Forming Polymers	Plasticizers	Others				
**Tablets**							
Caffeine, 5%, 10%, 20%	HPC SSL/Kollidon VA64/Kollicoat IR	Xylitol/PEG 4000	PEG4000, maltodextrin, dibasic calcium phosphate—pore formers	Honeycomb	8 × 16 × 4 mm	- Rapid release - Dissolution rate of drugs might be affected by just 10 °C variation in 3D printing temperature	[39]
Amiodarone hydrochloride, 20%	PEO (PolyoxN10) 40%	Glycerol 2%	D-sorbitol 37%—filler, colloidal anhydrous silica 1%—flow activator	Serpentine shape	-	- The influence of powder storage on batch reproducibility, water absorption was observed, which decreased powder flow and had a plasticising effect in the HME process - Content uniformity was proven	[48]
Placebo	Eudragit EPO 45%	Triethylcitrate 5%	Sodium stearyl fumarate—flow activator, TiO_2_ 1%—colouring agent, talc 49%—filler	Convex tablets	6 mm/8 mm/10 mm diameter	- Acceptability study on children aged 4 to 12 years old, 77% found them acceptable for a daily intake - Tablet size, followed by taste, texture, and finally smell, were the factors mentioned as important for the acceptability of a medicine	[66]
Hydrocortisone, 10–15%, 2.5 mg to 7.5 mg/tablet	65.45% Eudragit EPO	Triethylcitrate 4.55%	Sodium stearyl fumarate 4%, TiO_2_ 1%, Talc	Caplet	Diameter < 10 mm	- High drug-loading filaments were incompatible with 3D printing	[40]
Caffeine citrate 5%, 10%, 15%, 20%	HPC LF 60% –95%		HPMC K4M 20%—sustained release agent, Eudragit EPO 5–20%—taste masking agent	Doughnut	10 mm diameter	- API or Eudragit EPO concentration increase led to poor filament printability - The association of polymers demonstrated efficient taste masking	[47]
Tinidazole, 10–15%	Kollidon 25			Hexagonal, heart, pentagon, mickey-mouse, star, fish	12 mm/9 mm/6 mm/4 mm diameter and 3 mm height	- By incorporating the API into the polymer matrix, HME is considered to cover up bitter flavours by avoiding direct contact with the taste buds - Complex shapes and geometries were successfully prepared - No additional excipients, hence reducing the excipient toxicity risks	[52]
Sodium valproate, 10% and 30%	PEO 49-67-70-100%	PEG 6000 21–23–30%/PEG 35000 21 –23–30%		Scaffolds	13 × 19 × 2.5 mm (width × length × heights), decreased	- To improve water contact, the printed form was made using a grid pattern without a shell - Dosage forms designed to be administered in a liquid form after 15 min of dispersing in water	[54]
**Sprinklets**							
Celecoxib, 10%	80–90% Aquazol P500		TPGS 10%, SPL5%, surfactants	Doughnut, heart, star	<5 mm diameter, 2 mm height	- Surfactants also had a plasticizing effect and inhibited precipitation of the API during filament production - Supposed to result in improved swallowability - Celecoxib’s solubility and dissolution profile were greatly enhanced by the amorphous solid dispersion produced by HME	[44]
**Mini-tablets**							
Caffeine or Propranolol hydrochloride, 10%	HPMC (AFFINISOL™ HME 15LV)/ HPC (Klucel ELF)	PEG 6000 10%	Fumed silica 0.5%—flow regulator	Cylindrical	1.5 to 4 mm diameter	- Printed shape was found to be more irregular the smaller the diameter - Release was similar for both APIs when using HPC	[42]
Griseofulvin, 1–20%	84%/75%/65% HPC SL and 15% Kollicoat Protect	-	-	Cylindrical	1.5 mm diameter	- Ability to titrate doses in increments of 0.19 mg using single unit mini-tablets	[41]
Hydrocortisone, 20%, 2–8 mg/unit	PVP (Kollidon VA64), HPMC (AFFINISOL HME 15LV)	PEG 6000 10%, sorbitol 10% and triethylcitrate 3%	Red iron oxide—colouring agent	Waffle	31.79 to 132.4 mm^3^ volume	- Immediate release - Content and mass consistency were proven	[49]
Baclofen, 10%	PVA (Parteck MXP)	Sorbitol 10%	-	Caplet	5 mm/7.5 mm/10 mm diameter	-Printing at higher temperatures leads to high weight uniformity - From the tested patterns (shark fill, linear, hexagonal, diamond), the diamond infill led to the fastest disintegration	[50]
**Pastilles/Candy-like dosage form**							
Indomethacin, 20%, 25 mg/unit	HPMCAS 60% (AQOAT AS-MF)	PEG6000 20%		Bottle, heart, ring, bear	10 to 20 mm diameter	- The drug–polymer interaction through HME process allowed effective taste masking	[51]
**Chewable tablets**							
Diphenhydramine hydrochloride, 2.5%, 12.5 mg/unit	Klucel^®^ ELF 80%,		Gelucire 48/16 14.5%—surfactant, food colours 1%, strawberry flavour 1.1%, sucralose 0.9%,	Smurf, banana, cherry, palm tree	10 to 20 mm diameter	- Smurf design demonstrated the quickest API release	[43]
**Orodispersible films**							
Aripiprazole, 3.5%	PVA (Poval 4-88) 96.5%			Rectangle-shaped film	6 cm^2^	- The method of 3D printing is compared to electrospinning and solvent casting - Cast films have demonstrated the best stability; after storage, the 3D printed films showed improvements in tensile strength and Young’s modulus - Packaging must ensure stability for a long time	[53]
**Suppositories**							
Prednisolone sodium phosphate, 4%, 6–30 mg/unit	HPC (Klucel EF) 48%/73%/96%	Mannitol 23–48%		Torpedo-shaped	16 mm/21 mm/26 mm height	- Size was chosen to match the size of a 1.15 mL infant suppository mold - The mannitol-containing filament had high brittleness - Slow release with small mannitol ratios and immediate release with high mannitol ratios	[45]
Artesunate, 500 mg/unit	PVA		PEG3350, PEG1000			- Comparison of the 3D-printed products with fused suppositories with free API and API-loaded micelles - The suppository shell was 3D printed and filled with API-PEG mixtures	[56]
Cannabidiol	Placebo spring with thermoplastic urethane Shell with PVA	Shell with PEG	PEG3350, PEG1000			- PVA shell absorbs water, dissolves, and slowly releases the API - The spring was made by 3D printing thermoplastic urethane filaments, while the API-loaded shell was made using a 3D-printed metal mold - The hollow structure was designed to increase patient compliance	[57]

PEO, polyethylene oxide; HPC, hydroxypropylcellulose; PVA, polyvinyl alcohol; PVP, polyvinylpyrrolidone; PEG, polyethylene glycol.

**Table 3:** DPE 3D-printed paediatric dosage forms.

API Name, %, Dose/Unit	Excipients			Design	Size/Volume	Observations	Ref.
	Matrix-Forming Polymers	Plasticizers	Others				
**Tablets**							
Praziquantel, 35 or 50%, 100 mg API/unit	Kollidon VA 64 50%, 60%, 65%/ PEO 100000 60%/HPC ELF 95%	-	Span 20, Kolliphor SLS Fine 5%—surfactants	Cylindrical	10 mm diameter	- Efficient taste-masking - Printlets demonstrated stability after a three-month evaluation	[81]
Ibuprofen, 40%	Kollidon VA 64, Soluplus, or Eudragit EPO			Cylindrical	10 mm diameter	- Micro-extrusion technique enables a reduction in the total processing time of 3D printing as well as the waste of produced materials	[83]
Biotin, 5%	PEO 100000 60%/HPC ELF 95%	Mannitol 35% in the PEO pharma-ink		Capsule-shaped	2.7 mm diameter, 8.6 mm length	- Examined the implementation of a software-controlled, in-line analytical balance in a pharmaceutical multi-printhead DPE printer - Developed immediate release capsule-shaped tablets with PEO and extended-release ones with HPC	[84]
**Mini-tablets**							
Ritonavir or lopinavir, 25%, 65 mg API/unit	AQOAT^®^ LG (HPMCAS) 51.75%	22% PEG4000	0.75% magnesium stearate	Spherical	6 mm diameter	- Tablets were first prepared through HME and FDM, but this resulted in drug degradation - DPE was selected due to its decreased process temperature, 80 °C - Printed tablets were compared to commercially produced Kaletra; the mini-tablets had a zero-order release profile	[86]
Budesonide 0.59%	AFFINISOL HPMC HME 15 LV 41.84–75.44%	PEG6000 3.97–2.99%	Adjuvant blend 8.17–20% hydroxypropyl-β-cyclodextrin (HP-β-CD) 46.61%—solubility enhancer Eudragit FS 30D—coating agent	Cylindrical	5 mm diameter	- Targeted delivery of API to the colon was accomplished - Three-month stability evaluation demonstrated an absence of API degradation	[80]
Captopril	PVA 4-88 and AFFINISOL HPMC HME 15 LV			Cylindrical		- As a co-polymer, HPMC was added to improve drug encapsulation and guarantee structural integrity - By varying the infill, immediate release and sustained release tablets were created	[85]
**Mucoadhesive films**							
Clobetasol propionate, 0.20%, 125 µg/film	AFFINISOL HPMC HME 15 LV 0.35%	Polyox^™^ WSR N10 (PEO) 66.45–86.45%	HP-β-CD 3%—solubility enhancer Chitosan 10–30%—mucoadhesive	Cylindrical	20 mm diameter	- Retention of API inside the epithelium prevented systemic absorption - A progressive release of the drug over time was demonstrated - Three-month stability was established	[82]

HPMC, hydroxypropylcellulose; HPMCAS, hypromellose acetate succinate; PEG, polyethylene glycol; HP- β-CD, hydroxypropyl-β-cyclodextrin.

**Table 5:** Comparison between the three most used pharmaceutical 3D printing technologies.

	FDM	DPE	SSE
Advantages	- Most researched method - High design versatility - Good resolution - Printed products with good mechanical properties [149]	- Powder mixtures as input materials, no pre-processing - Lower thermal stress on API, compared to FDM [150] - Allows higher drug loads than FDM	- Low thermal stress on API, fit for thermosensitive APIs - Faster overall compared to other technologies - Products obtained via SSE accommodate wide dose ranges
Disadvantages	- Filament formulation must grant printability - Thermal treatment applied during extrusion and printing can lead to API degradation - API doses limited by filament printability [149]	- Materials are exposed to heat for a longer time than in FDM [149] - Powder properties (flowability) influence printability	- Lower resolution when compared to FDM and DPE [150] - Material viscosity must be controlled to have a printable feedstock [150] - Variable solidification time [150] -Solvent use
Method complexity	- Medium complexity - Three stages: powder mixing, hot-melt extrusion and printing	- Low complexity - Two stages: powder mixing and printing	- Medium complexity - Three stages: semisolid ink Preparation, printing and solidification (cooling/drying)
Costs	- Relatively low	- Medium	- medium to high
Required know-how	- Basic 3DP principles and design software knowledge - Hot-melt extrusion process and formulation principles - Mechanical characterisation and optimisation of filaments - Pharmaceutical characterization of 3DP dosage forms	- Basic 3DP principles and design software knowledge - Powder flow characterisation and optimisation - Pharmaceutical characterization of 3DP dosage forms	- Basic 3DP principles and design software knowledge - Understanding of the rheology of semisolid products - Cooling/drying process knowledge - Pharmaceutical characterisation of 3DP dosage forms
Suitability for printing paediatric dosage forms	- Suitable for customised sizes, shapes, and doses - Dose flexibility and accurate dose titration confirmed - Various paediatric-friendly dosage forms: tablets, mini-tablets, pastilles, chewable tablets, orodispersible dosage forms, chewable tablets, and suppositories - High stability	- Suitable for tablets, minitablets, and mucoadhesive films - Good stability for several months	- Highly suitable for chewable formulations, orodispersible dosage forms, tablets and mini-tablets
Translational challenges	- Need for industrially manufactured filaments and GMP scale-up [90] - Non-destructive and inline quality control demand - Few or no clinical implementations	- GMP-scale reproducibility needs a powder feed and flowability control for a consistent API distribution in printlets [79] - Establishing quality control methods	- Rheology and solidification have to be controlled for large scale production [151] - Establishing automated quality control methods
Clinical translation and Acceptability	- Used in early acceptability studies with placebo tablets with the highest acceptability score for torus geometry [152], and another study with an 83% overall acceptability, based on the evaluation of 30 participants [66]	- Limited clinical translation for children so far	- Hospital clinical trials for children have been conducted with success [122,103] and stability, human sensory evaluations were assessed in a hospital setting [121] - Established feasibility in a community pharmacy setting [153]
Development maturity level	Mid-stage	Emerging	Advanced (for community and hospital pharmacies)

## Data Availability

This article is based on a comprehensive literature review. No new data have been generated or analyzed beyond the literature search queries and the results obtained from the reviewed publications. All references and search strategies are detailed within the manuscript.

## Abbreviations

The following abbreviations are used in this manuscript, in this order:

3D	Three-dimensional
HME	Hot melt extrusion
FDM	Fused deposition modelling
DPE	Direct powder extrusion
SSE	Semi-solid extrusion
SLS	Selective laser sintering
BJ	Binder-jet
WHO	World Health Organization
APIs	Active pharmaceutical ingredients
ODTs	Orodispersible tablets
PAM	Pressure-assisted microsyringe
ODFs	Orodispersible films
Tm	Melting temperature
Tg	Glass transition temperature
STEP	Safety and Toxicity of Excipients for Paediatrics
GRAS	Generally Recognised as Safe
HPC	Hydroxypropylcellulose
HPMC	Hydroxypropylmetylcellulose
HPMC-AS	Hypromellose acetate succinate
BCS	Biopharmaceutical Classification System
PVP	Polyvinylpyrrolidone
PVA	Poly(vinyl) alcohol
PEO	Polyethylene oxide
PEG	Polyethylene glycol
TPGS	D-α-Tocopheryl PEG 1000 succinate
DPH	Diphenhydramine hydrochloride
FTIR	Fourier Transform Infrared Spectroscopy
CMS-Na	Sodium carboxymethyl starch
GABA	Gamma-aminobutyric acid
SSG	Sodium starch glycolate
FDA	Food and Drug Administration
CDI	Chewing Difficulty Index
DoD	Drop on demand
CIJ	Continuous inkjet
MW	Molecular weight
PBPK	Physiologically based pharmacokinetic
CJ-3DP	Colour-jet 3D printing
AIDS	Acquired Immune Deficiency Syndrome
NIR	Near-infrared
TRL	Technology Readiness Level
GMP	Good Manufacturing Practice

## References

[1] Lopez F.L., Ernest T.B., Tuleu C., Gul M.O. (2015). Formulation approaches to pediatric oral drug delivery: Benefits and limitations of current platforms. Expert. Opin. Drug Deliv..

[2] Sam T., Ernest T.B., Walsh J., Williams J.L. (2012). A benefit/risk approach towards selecting appropriate pharmaceutical dosage forms—An application for paediatric dosage form selection. Int. J. Pharm..

[3] Matsui D. (2007). Current Issues in Pediatric Medication Adherence. Pediatr. Drugs.

[4] Walsh J., Cram A., Woertz K., Breitkreutz J., Winzenburg G., Turner R., Tuleu C. (2014). Playing hide and seek with poorly tasting paediatric medicines: Do not forget the excipients. Adv. Drug Deliv. Rev..

[5] Belayneh A., Tadese E., Molla F. (2020). Safety and biopharmaceutical challenges of excipients in off-label pediatric formulations. Int. J. Gen. Med..

[6] Zajicek A., Fossler M.J., Barrett J.S., Worthington J.H., Ternik R., Charkoftaki G., Lum S., Breitkreutz J., Baltezor M., Macheras P. (2013). A report from the pediatric formulations task force: Perspectives on the state of child-friendly oral dosage forms. AAPS J..

[7] Dumpa N., Butreddy A., Wang H., Komanduri N., Bandari S., Repka M.A. (2021). 3D printing in personalized drug delivery: An overview of hot-melt extrusion-based fused deposition modeling. Int. J. Pharm..

[8] Madla C.M., Trenfield S.J., Goyanes A., Gaisford S., Basit A.W., Basit A.W., Gaisford S. (2018). 3D Printing Technologies, Implementation and Regulation: An Overview. 3D Printing of Pharmaceuticals.

[9] Ayyoubi S., Ruijgrok L., van der Kuy H., ten Ham R., Thielen F. (2025). What Does Pharmaceutical 3D Printing Cost? A Framework and Case Study with Hydrocortisone for Adrenal Insufficiency. Pharmacoecon. Open.

[10] McCloskey A.P., Bracken L., Vasey N., Ehtezazi T. (2023). 3D printing—An alternative strategy for pediatric medicines. Expert. Rev. Clin. Pharmacol..

[11] Ahmed M., Tomlin S., Tuleu C., Garfield S. (2024). Real-World Evidence of 3D Printing of Personalised Paediatric Medicines and Evaluating Its Potential in Children with Cancer: A Scoping Review. Pharmaceutics.

[12] Preis M., Öblom H. (2017). 3D-Printed Drugs for Children-Are We Ready Yet?. AAPS PharmSciTech.

[13] Lafeber I., Ruijgrok E.J., Guchelaar H.J., Schimmel K.J.M. (2022). 3D Printing of Pediatric Medication: The End of Bad Tasting Oral Liquids?-A Scoping Review. Pharmaceutics.

[14] Racaniello G.F., Silvestri T., Pistone M., D’Amico V., Arduino I., Denora N., Lopedota A.A. (2024). Innovative Pharmaceutical Techniques for Paediatric Dosage Forms: A Systematic Review on 3D Printing, Prilling/Vibration and Microfluidic Platform. J. Pharm. Sci..

[15] Ianno V., Vurpillot S., Prillieux S., Espeau P. (2024). Pediatric Formulations Developed by Extrusion-Based 3D Printing: From Past Discoveries to Future Prospects. Pharmaceutics.

[16] Quodbach J., Bogdahn M., Breitkreutz J., Chamberlain R., Eggenreich K., Elia A.G., Gottschalk N., Gunkel-Grabole G., Hoffmann L., Kapote D. (2022). Quality of FDM 3D Printed Medicines for Pediatrics: Considerations for Formulation Development, Filament Extrusion, Printing Process and Printer Design. Ther. Innov. Regul. Sci..

[17] Tong H., Zhang J., Ma J., Zhang J. (2024). Perspectives on 3D printed personalized medicines for pediatrics. Int. J. Pharm..

[18] Tegegne A.M., Ayenew K.D., Selam M.N. (2024). Review on Recent Advance of 3DP-Based Pediatric Drug Formulations. Biomed. Res. Int..

[19] Lu H., Rosenbaum S. (2014). Developmental pharmacokinetics in pediatric populations. J. Pediatr. Pharmacol. Ther..

[20] Mu Y., Zhao L., Shen L. (2023). Medication adherence and pharmaceutical design strategies for pediatric patients: An overview. Drug Discov. Today.

[21] Zahn J., Hoerning A., Trollmann R., Rascher W., Neubert A. (2020). Manipulation of medicinal products for oral administration to paediatric patients at a german university hospital: An observational study. Pharmaceutics.

[22] Binson G., Sanchez C., Waton K., Chanat A., Maio M.D., Beuzit K., Dupuis A. (2021). Accuracy of dose administered to children using off-labelled or unlicensed oral dosage forms. Pharmaceutics.

[23] Siafaka P., Ipekci E., Caglar E.Ş., Ustundag Okur N., Buyukkayhan D. (2021). Current status of pediatric formulations for chronic and acute children’ diseases: Applications and future perspectives. Medeni. Med. J..

[24] Batchelor H.K., Marriott J.F. (2015). Formulations for children: Problems and solutions. Br. J. Clin. Pharmacol..

[25] Ruiz F., Nunn A., Gill A., Clapham D., Fotaki N., Salunke S., Cram A., O’Brien F. (2023). A review of paediatric injectable drug delivery to inform the study of product acceptability—An introduction. Eur. J. Pharm. Biopharm..

[26] Delgado-Charro M.B., Guy R.H. (2014). Effective use of transdermal drug delivery in children. Adv. Drug Deliv. Rev..

[27] Mistry P., Batchelor H. (2017). Evidence of acceptability of oral paediatric medicines: A review. J. Pharm. Pharmacol..

[28] Gaikwad S.S., Morales J.O., Lande N.B., Catalán-Figueroa J., Laddha U.D., Kshirsagar S.J. (2024). Exploring paediatric oral suspension development: Challenges, requirements, and formulation advancements. Int. J. Pharm..

[29] Michele T.M., Knorr B., Vadas E.B., Reiss T.F. (2002). Safety of chewable tablets for children. J. Asthma.

[30] Rai A., Rawat S.S., Rathi R., Raina D., Odeku O.A., Singh I. (2023). Mucoadhesive Drug Delivery Systems for Pediatric and Geriatric Patients. Fabad J. Pharm. Sci..

[31] Tai J., Han M., Lee D., Park I.H., Lee S.H., Kim T.H. (2022). Different Methods and Formulations of Drugs and Vaccines for Nasal Administration. Pharmaceutics.

[32] Palmer E.A. (1986). How Safe are Ocular Drugs in Pediatrics?. Ophthalmology.

[33] Magdy M., Elmowafy E., Elassal M., Ishak R.A.H. (2022). Localized drug delivery to the middle ear: Recent advances and perspectives for the treatment of middle and inner ear diseases. J. Drug Deliv. Sci. Technol..

[34] Kwok P.C.L., Chan H.K. (2014). Delivery of inhalation drugs to children for asthma and other respiratory diseases. Adv. Drug Deliv. Rev..

[35] Hanning S.M., Walker E., Sutcliffe E., Tuleu C. (2020). The rectal route of medicine administration for children: Let’s get to the bottom of it!. Eur. J. Pharm. Biopharm..

[36] Pires L.R., Vinayakumar K.B., Turos M., Miguel V., Gaspar J. (2019). A perspective on microneedle-based drug delivery and diagnostics in paediatrics. J. Pers. Med..

[37] Tambe S., Jain D., Agarwal Y., Amin P. (2021). Hot-melt extrusion: Highlighting recent advances in pharmaceutical applications. J. Drug Deliv. Sci. Technol..

[38] Goyanes A., Buanz A.B.M., Hatton G.B., Gaisford S., Basit A.W. (2015). 3D printing of modified-release aminosalicylate (4-ASA and 5-ASA) tablets. Eur. J. Pharm. Biopharm..

[39] Fanous M., Gold S., Hirsch S., Ogorka J., Imanidis G. (2020). Development of immediate release (IR) 3D-printed oral dosage forms with focus on industrial relevance. Eur. J. Pharm. Sci..

[40] Yang T.L., Stogiannari M., Janeczko S., Khoshan M., Lin Y., Isreb A., Habashy R., Giebułtowicz J., Peak M., Alhnan M.A. (2023). Towards point-of-care manufacturing and analysis of immediate-release 3D printed hydrocortisone tablets for the treatment of congenital adrenal hyperplasia. Int. J. Pharm..

[41] Gorkem Buyukgoz G., Kossor C.G., Ji S., Guvendiren M., Davé R.N. (2022). Dose Titration of Solid Dosage Forms via FDM 3D-Printed Mini-Tablets. Pharmaceutics.

[42] Krause J., Müller L., Sarwinska D., Seidlitz A., Sznitowska M., Weitschies W. (2021). 3D printing of mini tablets for pediatric use. Pharmaceuticals.

[43] Tabriz A.G., Fullbrook D.H.G., Vilain L., Derrar Y., Nandi U., Grau C., Morales A., Hooper G., Hiezl Z., Douroumis D. (2021). Personalised tasted masked chewable 3d printed fruit-chews for paediatric patients. Pharmaceutics.

[44] Patel H., Raje V., Maczko P., Patel K. (2024). Application of 3D printing technology for the development of dose adjustable geriatric and pediatric formulation of celecoxib. Int. J. Pharm..

[45] Kocabas L.I., Ayyoubi S., Tajqurishi M., Quodbach J., Vermonden T., Kok R.J. (2024). 3D-printed prednisolone phosphate suppositories with tunable dose and rapid release for the treatment of inflammatory bowel disease. Int. J. Pharm..

[46] World Health Organization Model List of Essential Medicines for Children 9th List (2023). https://iris.who.int/bitstream/handle/10665/371091/WHO-MHP-HPS-EML-2023.03-eng.pdf?sequence=1.

[47] Wang H., Dumpa N., Bandari S., Durig T., Repka M.A. (2020). Fabrication of Taste-Masked Donut-Shaped Tablets Via Fused Filament Fabrication 3D Printing Paired with Hot-Melt Extrusion Techniques. AAPS PharmSciTech.

[48] Roulon S., Soulairol I., Lavastre V., Payre N., Cazes M., Delbreilh L., Alié J. (2021). Production of reproducible filament batches for the fabrication of 3d printed oral forms. Pharmaceutics.

[49] Parulski C., Bya L.A., Goebel J., Servais A.C., Lechanteur A., Evrard B. (2023). Development of 3D printed mini-waffle shapes containing hydrocortisone for children’s personalized medicine. Int. J. Pharm..

[50] Palekar S., Kumar P., Mishra S.M., Kipping T., Patel K. (2019). Application of 3D printing technology and quality by design approach for development of age-appropriate pediatric formulation of baclofen. Int. J. Pharm..

[51] Scoutaris N., Ross S.A., Douroumis D. (2018). 3D Printed “Starmix” Drug Loaded Dosage Forms for Paediatric Applications. Pharm. Res..

[52] Pawar A., Karanwad T., Banerjee S. (2024). 3D printed tinidazole tablets coupled with melt-extrusion techniques for formulating child friendly medicines. Eur. J. Pharm. Biopharm..

[53] Łyszczarz E., Brniak W., Szafraniec-Szczęsny J., Majka T.M., Majda D., Zych M., Pielichowski K., Jachowicz R. (2021). The impact of the preparation method on the properties of orodispersible films with aripiprazole: Electrospinning vs. casting and 3D printing methods. Pharmaceutics.

[54] Monteil M., Sanchez-Ballester N.M., Aubert A., Gimello O., Begu S., Soulairol I. (2025). HME coupled with FDM 3D printing of a customized oral solid form to treat pediatric epilepsy. Int. J. Pharm..

[55] Ferreira M., Lopes C.M., Gonçalves H., Pinto J.F., Catita J. (2022). Personalised Esomeprazole and Ondansetron 3D Printing Formulations in Hospital Paediatric Environment: I-Pre-Formulation Studies. Appl. Sci..

[56] Persaud S., Eid S., Swiderski N., Serris I., Cho H. (2020). Preparations of rectal suppositories containing artesunate. Pharmaceutics.

[57] Wei M., Liu D., Xie H., Sun Y., Fang Y., Du L., Jin Y. (2025). 3D-printed cannabidiol hollow suppositories for treatment of epilepsy. Int. J. Pharm..

[58] Ahirwar K., Shukla R. (2023). Preformulation Studies: A Versatile Tool in Formulation Design. Drug Formulation Design.

[59] EMA Guideline on Pharmaceutical Development of Medicines for Paediatric Use. https://www.tga.gov.au/sites/default/files/2024-07/Guidelineonpharmaceuticaldevelopmentofmedicinesforpaediatric%20use%20.pdf.

[60] Kristensen H.G. (2012). WHO guideline development of paediatric medicines: Points to consider in pharmaceutical development. Int. J. Pharm..

[61] STEP Database. https://step-db.ucl.ac.uk/eupfi/appDirectLink.do?appFlag=login.

[62] Salunke S., Giacoia G., Tuleu C. (2012). The STEP (safety and toxicity of excipients for paediatrics) database. Part 1-A need assessment study. Int. J. Pharm..

[63] Solanki N.G., Tahsin M., Shah A.V., Serajuddin A.T.M. (2018). Formulation of 3D Printed Tablet for Rapid Drug Release by Fused Deposition Modeling: Screening Polymers for Drug Release, Drug-Polymer Miscibility and Printability. J. Pharm. Sci..

[64] Couți N., Porfire A., Iovanov R., Crișan A.G., Iurian S., Casian T., Tomuță I. (2024). Polyvinyl Alcohol, a Versatile Excipient for Pharmaceutical 3D Printing. Polymers.

[65] Parteck^®^ MXP Technical Information. https://www.sigmaaldrich.com/deepweb/assets/sigmaaldrich/product/documents/203/518/parteck-mxp-techinfo-141464-mk.pdf.

[66] Bracken L., Habashy R., McDonough E., Wilson F., Shakeshaft J., Ohia U., Garcia-Sorribes T., Isreb A., Alhnan M.A., Peak M. (2022). Creating Acceptable Tablets 3D (CAT 3D): A Feasibility Study to Evaluate the Acceptability of 3D Printed Tablets in Children and Young People. Pharmaceutics.

[67] Eudragit E PO. https://www.pharmaexcipients.com/product/eudragit-e-po/?attachment_id=201953&download_file=2ife21drt18ev.

[68] Alhijjaj M., Belton P., Qi S. (2016). An investigation into the use of polymer blends to improve the printability of and regulate drug release from pharmaceutical solid dispersions prepared via fused deposition modeling (FDM) 3D printing. Eur. J. Pharm. Biopharm..

[69] Gelucire 48/16. https://www.pharmaexcipients.com/wp-content/uploads/2020/03/Gelucire-48-16_solubility-and-bioavailability-enhancer-from-Gattefosse.pdf.

[70] Januskaite P., Xu X., Ranmal S.R., Gaisford S., Basit A.W., Tuleu C., Goyanes A. (2020). I spy with my little eye: A paediatric visual preferences survey of 3d printed tablets. Pharmaceutics.

[71] Kozarewicz P. (2014). Regulatory perspectives on acceptability testing of dosage forms in children. Int. J. Pharm..

[72] Mitra B., Thool P., Meruva S., Aycinena J.A., Li J., Patel J., Patel K., Agarwal A., Karki S., Bowen W. (2020). Decoding the small size challenges of mini-tablets for enhanced dose flexibility and micro-dosing. Int. J. Pharm..

[73] Klingmann V., Spomer N., Lerch C., Stoltenberg I., Frömke C., Bosse H.M., Breitkreutz J., Meissner T. (2013). Favorable acceptance of mini-tablets compared with syrup: A randomized controlled trial in infants and preschool children. J. Pediatr..

[74] Lura A., Tardy G., Kleinebudde P., Breitkreutz J. (2020). Tableting of mini-tablets in comparison with conventionally sized tablets: A comparison of tableting properties and tablet dimensions. Int. J. Pharm. X.

[75] Kukkar V., Anand V., Kataria M., Gera M., Choudhury P.K. (2008). Mixing and formulation of low dose drugs: Underlying problems and solutions. Thai J. Pharm. Sci..

[76] Cilurzo F., Musazzi U.M., Franzé S., Selmin F., Minghetti P. (2018). Orodispersible dosage forms: Biopharmaceutical improvements and regulatory requirements. Drug Discov. Today.

[77] Mennella J.A., Spector A.C., Reed D.R., Coldwell S.E. (2013). The Bad Taste of Medicines: Overview of Basic Research on Bitter Taste. Clin. Ther..

[78] Jannin V., Lemagnen G., Gueroult P., Larrouture D., Tuleu C. (2014). Rectal route in the 21st Century to treat children. Adv. Drug Deliv. Rev..

[79] Aguilar-de-Leyva Á., Casas M., Ferrero C., Linares V., Caraballo I. (2024). 3D Printing Direct Powder Extrusion in the Production of Drug Delivery Systems: State of the Art and Future Perspectives. Pharmaceutics.

[80] Pistone M., Racaniello G.F., Rizzi R., Iacobazzi R.M., Arduino I., Lopalco A., Lopedota A.A., Denora N. (2023). Direct cyclodextrin based powder extrusion 3D printing of budesonide loaded mini-tablets for the treatment of eosinophilic colitis in paediatric patients. Int. J. Pharm..

[81] Boniatti J., Januskaite P., da Fonseca L.B., Viçosa A.L., Amendoeira F.C., Tuleu C., Basit A.W., Goyanes A., Ré M.I. (2021). Direct powder extrusion 3d printing of praziquantel to overcome neglected disease formulation challenges in paediatric populations. Pharmaceutics.

[82] Racaniello G.F., Pistone M., Meazzini C., Lopedota A., Arduino I., Rizzi R., Lopalco A., Musazzi U.M., Cilurzo F., Denora N. (2023). 3D printed mucoadhesive orodispersible films manufactured by direct powder extrusion for personalized clobetasol propionate based paediatric therapies. Int. J. Pharm..

[83] Tabriz A.G., Nandi U., Scoutaris N., Sanfo K., Alexander B., Gong Y., Hui H.W., Kumar S., Douroumis D. (2022). Personalised paediatric chewable Ibuprofen tablets fabricated using 3D micro-extrusion printing technology. Int. J. Pharm..

[84] Mora-Castaño G., Rodríguez-Pombo L., Carou-Senra P., Januskaite P., Rial C., Bendicho-Lavilla C., Couce M.L., Millán-Jiménez M., Caraballo I., Basit A.W. (2025). Optimising 3D printed medications for rare diseases: In-line mass uniformity testing in direct powder extrusion 3D printing. Int. J. Pharm..

[85] Totaro M., Racaniello G.F., Lopalco A., Lopedota A.A., Denora N. (2025). Development of 3D-Printed Captopril Mini-Tablets with customized release profiles for paediatric hypertension therapy. Int. J. Pharm..

[86] Malebari A.M., Kara A., Khayyat A.N., Mohammad K.A., Serrano D.R. (2022). Development of Advanced 3D-Printed Solid Dosage Pediatric Formulations for HIV Treatment. Pharmaceuticals.

[87] Ozon E.A., Sarbu I., Popovici V., Mitu M.A., Musuc A.M., Karampelas O., Velescu B.S. (2023). Three-Dimensional Printing Technologies in Oral Films Manufacturing—A Minireview. Processes.

[88] Neagu O.M., Ghitea T., Marian E., Vlase L., Vlase A.M., Ciavoi G., Fehér P., Pallag A., Bácskay I., Nemes D. (2023). Formulation and Characterization of Mucoadhesive Polymeric Films Containing Extracts of Taraxaci Folium and Matricariae Flos. Molecules.

[89] El Aita I., Rahman J., Breitkreutz J., Quodbach J. (2020). 3D-Printing with precise layer-wise dose adjustments for paediatric use via pressure-assisted microsyringe printing. Eur. J. Pharm. Biopharm..

[90] Wang S., Chen X., Han X., Hong X., Li X., Zhang H., Li M., Wang Z., Zheng A. (2023). A Review of 3D Printing Technology in Pharmaceutics: Technology and Applications, Now and Future. Pharmaceutics.

[91] Seoane-Viaño I., Januskaite P., Alvarez-Lorenzo C., Basit A.W., Goyanes A. (2021). Semi-solid extrusion 3D printing in drug delivery and biomedicine: Personalised solutions for healthcare challenges. J. Control. Release.

[92] Johannesson J., Khan J., Hubert M., Teleki A., Bergström C.A.S. (2021). 3D-printing of solid lipid tablets from emulsion gels. Int. J. Pharm..

[93] Lafeber I., Tichem J.M., Ouwerkerk N., van Unen A.D., van Uitert J.J.D., Bijleveld-Olierook H.C.M., Kweekel D.M., Zaal W.M., Le Brun P.P.H., Guchelaar H.J. (2021). 3D printed furosemide and sildenafil tablets: Innovative production and quality control. Int. J. Pharm..

[94] Liu L., Fu K., Hong S., Wang Z., Mo M., Li S., Yu Y., Chen J., Chen J., Zeng W. (2023). Improving the quality and clinical efficacy of subdivided levothyroxine sodium tablets by 3D printing technology. J. Drug Deliv. Sci. Technol..

[95] Tagami T., Ito E., Kida R., Hirose K., Noda T., Ozeki T. (2021). 3D printing of gummy drug formulations composed of gelatin and an HPMC-based hydrogel for pediatric use. Int. J. Pharm..

[96] Han X., Kang D., Liu B., Zhang H., Wang Z., Gao X., Zheng A. (2022). Feasibility of developing hospital preparation by semisolid extrusion 3D printing: Personalized amlodipine besylate chewable tablets. Pharm. Dev. Technol..

[97] Liang E., Wang Z., Li X., Wang S., Han X., Chen D., Zheng A. (2023). 3D Printing Technology Based on Versatile Gelatin-Carrageenan Gel System for Drug Formulations. Pharmaceutics.

[98] Zhu C., Tian Y., Zhang E., Gao X., Zhang H., Liu N., Han X., Sun Y., Wang Z., Zheng A. (2022). Semisolid Extrusion 3D Printing of Propranolol Hydrochloride Gummy Chewable Tablets: An Innovative Approach to Prepare Personalized Medicine for Pediatrics. AAPS PharmSciTech.

[99] Bahman M., Sandler Topelius N., Viitala T. (2025). Semi-solid extruded tablets for personalized pediatric use: Development, Quality control and In-Vitro Assessment of Enteral Tube Administration. Eur. J. Pharm. Sci..

[100] Roostar K., Meos A., Laidmäe I., Aruväli J., Räikkönen H., Peltonen L., Airaksinen S., Topelius N.S., Heinämäki J., Paaver U. (2025). Towards a Customized Oral Drug Therapy for Pediatric Applications: Chewable Propranolol Gel Tablets Printed by an Automated Extrusion-Based Material Deposition Method. Pharmaceutics.

[101] Veselý M., Záruba D., Elbl J. (2025). Development of 3D-Printed Chewable Gummy Tablets with Adjustable Ondansetron Content for the Treatment of Pediatric Patients. Pharmaceutics.

[102] Herrada-Manchón H., Rodríguez-González D., Alejandro Fernández M., Suñé-Pou M., Pérez-Lozano P., García-Montoya E., Aguilar E. (2020). 3D printed gummies: Personalized drug dosage in a safe and appealing way. Int. J. Pharm..

[103] Goyanes A., Madla C.M., Umerji A., Duran Piñeiro G., Giraldez Montero J.M., Lamas Diaz M.J., Gonzalez Barcia M., Taherali F., Sánchez-Pintos P., Couce M.L. (2019). Automated therapy preparation of isoleucine formulations using 3D printing for the treatment of MSUD: First single-centre, prospective, crossover study in patients. Int. J. Pharm..

[104] Karavasili C., Gkaragkounis A., Moschakis T., Ritzoulis C., Fatouros D.G. (2020). Pediatric-friendly chocolate-based dosage forms for the oral administration of both hydrophilic and lipophilic drugs fabricated with extrusion-based 3D printing. Eur. J. Pharm. Sci..

[105] Rycerz K., Stepien K.A., Czapiewska M., Arafat B.T., Habashy R., Isreb A., Peak M., Alhnan M.A. (2019). Embedded 3D Printing of Novel Bespoke Soft Dosage Form Concept for Pediatrics. Pharmaceutics.

[106] Chachlioutaki K., Karavasili C., Mavrokefalou E.E., Gioumouxouzis C.I., Ritzoulis C., Fatouros D.G. (2022). Quality control evaluation of paediatric chocolate-based dosage forms: 3D printing vs mold-casting method. Int. J. Pharm..

[107] Rouaz-El Hajoui K., Herrada-Manchón H., Rodríguez-González D., Fernández M.A., Aguilar E., Suñé-Pou M., Nardi-Ricart A., Pérez-Lozano P., García-Montoya E. (2023). Pellets and gummies: Seeking a 3D printed gastro-resistant omeprazole dosage for paediatric administration. Int. J. Pharm..

[108] Suárez-González J., Magariños-Triviño M., Díaz-Torres E., Cáceres-Pérez A.R., Santoveña-Estévez A., Fariña J.B. (2021). Individualized orodispersible pediatric dosage forms obtained by molding and semi-solid extrusion by 3D printing: A comparative study for hydrochlorothiazide. J. Drug Deliv. Sci. Technol..

[109] Eduardo D.T., Ana S.E., José B.F. (2021). A micro-extrusion 3D printing platform for fabrication of orodispersible printlets for pediatric use. Int. J. Pharm..

[110] Hu J., Fitaihi R., Abukhamees S., Abdelhakim H.E. (2023). Formulation and Characterisation of Carbamazepine Orodispersible 3D-Printed Mini-Tablets for Paediatric Use. Pharmaceutics.

[111] Sjöholm E., Sandler N. (2019). Additive manufacturing of personalized orodispersible warfarin films. Int. J. Pharm..

[112] Shokraneh F., Filppula A.M., Tornio A., Aruväli J., Paaver U., Topelius N.S. (2025). Automated extrusion-based dispensing: Personalized dosing and quality control of clopidogrel tablets for pediatric care. Eur. J. Pharm. Sci..

[113] Protopapa C., Siamidi A., Kolipaka S.S., Junqueira L.A., Douroumis D., Vlachou M. (2024). In Vitro Profile of Hydrocortisone Release from Three-Dimensionally Printed Paediatric Mini-Tablets. Pharmaceutics.

[114] Bernhardt M.B., Shokraneh F., Hrizanovska L., Lahtinen J., Brasher C.A., Sandler N. (2025). Automated 3D Printing-Based Non-Sterile Compounding Technology for Pediatric Corticosteroid Dosage Forms in a Health System Pharmacy Setting. Pharmaceutics.

[115] Santamaría K.J., Anaya B.J., Lalatsa A., González-Barranco P., Cantú-Cárdenas L., Serrano D.R. (2024). Engineering 3D Printed Gummies Loaded with Metformin for Paediatric Use. Gels.

[116] Chachlioutaki K., Li X., Koltsakidis S., Abdelhakim H.E., Bouropoulos N., Tzetzis D., Karavasili C., Fatouros D.G. (2025). How sugar types and fabrication methods affect palatability in paediatric-friendly oromucosal pullulan films of chlorpromazine hydrochloride. Carbohydr. Polym..

[117] Imbriano A., Fratini C., Bondi G., D’Abbrunzo I., Bertoni S., Tiboni M., Abruzzo A., Hasa D., Pagano C., Casettari L. (2025). 3D-printed chewable gummy tablets: A new tool for oral amoxicillin administration in paediatric population. Int. J. Pharm..

[118] Moreira A.d.O.E., Neta L.M.S.A., Pietroluongo M., Matos A.P.d.S., Correa B.B., Ortiz B.H., Guimarães A.d.S., Nele M., Santos C.M., Fai A.E.C. (2025). Three-Dimensional-Printed Isoniazid Chewable Gels for On-Demand Latent Tuberculosis Treatment in Children. Pharmaceutics.

[119] Dadkhah A., Gutowski T., Wansing E.M., von Hugo A., Woessmann W., Winkler B., Franke G., Baehr M., Langebrake C. (2025). Development of 3D printed dexamethasone chewable tablets for prophylaxis of chemotherapy-induced nausea and vomiting in children. Int. J. Pharm. X.

[120] Paccione N., Guarnizo-Herrero V., Navarro-Alvarez A., Scaini D., Larrarte E., Pedraz J.L. (2025). Development of personalized dexamethasone orodispersible solid oral dosage forms by semisolid extrusion 3D printing. Int. J. Pharm..

[121] Stoops M., Do B., Ramos S., Tan B.X., Sheng Chua N.Y., Mazet R., Guiblin N., Michelet A., Flynn S., Abbou S. (2025). Clinical implementation of a paediatric 3D-printed combination of Sulfamethoxazole and Trimethoprim. Int. J. Pharm..

[122] Rodríguez-Pombo L., de Castro-López M.J., Sánchez-Pintos P., Giraldez-Montero J.M., Januskaite P., Duran-Piñeiro G., Dolores Bóveda M., Alvarez-Lorenzo C., Basit A.W., Goyanes A. (2024). Paediatric clinical study of 3D printed personalised medicines for rare metabolic disorders. Int. J. Pharm..

[123] Strand D.S., Kim D., Peura D.A. (2017). 25 Years of Proton Pump Inhibitors: A Comprehensive Review. Gut Liver.

[124] CAPTEX^®^ MEDIUM CHAIN TRIGLYCERIDES. https://www.abiteccorp.com/en/product-repository/captex-medium-chain-triglycerides/.

[125] Maisine® CC. https://www.pharmaexcipients.com/wp-content/uploads/2020/03/Maisine-CC_gattefosse-pharmaceutical-oil-for-solubility-and-bioavailability-enhancement.pdf.

[126] Gelucire^®^ 44/14. https://www.gattefosse.com/pharmaceuticals/product-finder/gelucire-4414.

[127] CAPMUL^®^ MONO AND DIGLYCERIDES. https://www.abiteccorp.com/en/product-repository/capmul-mono-and-diglycerides/.

[128] Krämer J., Gajendran J., Guillot A., Barakat A. (2019). Chewable Oral Drug Products. In Vitro Drug Release Testing of Special Dosage Forms.

[129] Parhi R. (2021). A review of three-dimensional printing for pharmaceutical applications: Quality control, risk assessment and future perspectives. J. Drug Deliv. Sci. Technol..

[130] Hirshfield L., Giridhar A., Taylor L.S., Harris M.T., Reklaitis G.V. (2014). Dropwise additive manufacturing of pharmaceutical products for solvent-based dosage forms. J. Pharm. Sci..

[131] Konta A.A., García-Piña M., Serrano D.R. (2017). Personalised 3D printed medicines: Which techniques and polymers are more successful?. Bioengineering.

[132] Sundarkumar V., Wang W., Nagy Z., Reklaitis G. (2023). Manufacturing pharmaceutical mini-tablets for pediatric patients using drop-on-demand printing. Int. J. Pharm..

[133] Cui M., Pan H., Fang D., Sun H., Qiao S., Pan W. (2021). Exploration and evaluation of dynamic dose-control platform for pediatric medicine based on Drop-on-Powder 3D printing technology. Int. J. Pharm..

[134] Sundarkumar V., Wang W., Mills M., Oh S.W., Nagy Z., Reklaitis G. (2024). Developing a Modular Continuous Drug Product Manufacturing System with Real Time Quality Assurance for Producing Pharmaceutical Mini-Tablets. J. Pharm. Sci..

[135] Li Z.R., Wang C.Y., Lin W.W., Chen Y.T., Liu X.Q., Jiao Z. (2023). Handling Delayed or Missed Dose of Antiseizure Medications: A Model-Informed Individual Remedial Dosing. Neurology.

[136] Li X., Liang E., Hong X., Han X., Li C., Wang Y., Wang Z., Zheng A. (2022). In vitro and in vivo bioequivalence study of 3d-printed instant-dissolving levetiracetam tablets and subsequent personalized dosing for chinese children based on physiological pharmacokinetic modeling. Pharmaceutics.

[137] Tang Z., Chen X., Hong X., Han X., Li J., Duan S., Wu J., Wang Z., Zheng A. (2025). 3D printing personalized orally disintegrating tablets with complex structures for the treatment of special populations. Int. J. Pharm..

[138] Alhnan M.A., Okwuosa T.C., Sadia M., Wan K.W., Ahmed W., Arafat B. (2016). Emergence of 3D Printed Dosage Forms: Opportunities and Challenges. Pharm. Res..

[139] Wang Z., Han X., Chen R., Li J., Gao J., Zhang H., Liu N., Gao X., Zheng A. (2021). Innovative color jet 3D printing of levetiracetam personalized paediatric preparations. Asian J. Pharm. Sci..

[140] Iwanaga S., Arai K., Nakamura M. (2015). Inkjet bioprinting. Essentials of 3D Biofabrication and Translation.

[141] Kondiah P.P.D., Rants T.A., Mdanda S. (2022). An Oral 3D Printed PLGA-Tocopherol PEG Succinate. Biomedicines.

[142] Charoo N.A., Barakh Ali S.F., Mohamed E.M., Kuttolamadom M.A., Ozkan T., Khan M.A., Rahman Z. (2020). Selective laser sintering 3D printing–an overview of the technology and pharmaceutical applications. Drug Dev. Ind. Pharm..

[143] Gueche Y.A., Sanchez-Ballester N.M., Cailleaux S., Bataille B., Soulairol I. (2021). Selective laser sintering (Sls), a new chapter in the production of solid oral forms (sofs) by 3d printing. Pharmaceutics.

[144] Tarekegn K., Lonsako S., Hyattsville M.D., Ghidey F. (2015). Role of Continuity of Care in HIV/AIDS Treatment and Care Program in Ethiopia. Open Forum Infect. Dis..

[145] Kayalar C., Rahman Z., Mohamed E.M., Dharani S., Khuroo T., Helal N., Kuttolamadom M.A., Khan M.A. (2023). Preparation and Characterization of 3D-Printed Dose-Flexible Printlets of Tenofovir Disoproxil Fumarate. AAPS PharmSciTech.

[146] Pansare S.J., Kayalar C., Shaikh R., Dongala B.P., Thota S.K., Kuttolamadom M.A., Rahman Z., Khan M.A. (2025). Design of SLS 3D-printed pediatric combination printlets of lamivudine and tenofovir disoproxil fumarate by understanding the impact of formulation and process variables on flow, spectral, thermal and performance characteristics. Int. J. Pharm..

[147] Funk N.L., Januskaite P., Beck R.C.R., Basit A.W., Goyanes A. (2024). 3D printed dispersible efavirenz tablets: A strategy for nasogastric administration in children. Int. J. Pharm..

[148] Kayalar C., Pansare S.J., Sibhat G., Kuttolamadom M., Rahman Z., Khan M.A. (2025). Development and Characterization of Printlets of Lamivudine for Pediatric Patients Using Selective Laser Sintering. Pharmaceuticals.

[149] Paccione N., Guarnizo-Herrero V., Ramalingam M., Larrarte E., Pedraz J.L. (2024). Application of 3D printing on the design and development of pharmaceutical oral dosage forms. J. Control Release.

[150] Seoane-Viaño I., Otero-Espinar F.J., Goyanes Á. (2021). 3D printing of pharmaceutical products. Additive Manufacturing.

[151] Huanbutta K., Burapapadh K., Sriamornsak P., Sangnim T. (2023). Practical Application of 3D Printing for Pharmaceuticals in Hospitals and Pharmacies. Pharmaceutics.

[152] Goyanes A., Scarpa M., Kamlow M., Gaisford S., Basit A.W., Orlu M. (2017). Patient acceptability of 3D printed medicines. Int. J. Pharm..

[153] Rodríguez-Maciñeiras X., Bendicho-Lavilla C., Rial C., Garba-Mohammed K., Worsley A., Díaz-Torres E., Orive-Martínez C., Orive-Mayor Á., Basit A.W., Alvarez-Lorenzo C. (2025). Advancing medication compounding: Use of a pharmaceutical 3D printer to auto-fill minoxidil capsules for dispensing to patients in a community pharmacy. Int. J. Pharm..

[154] Seoane-Viaño I., Xu X., Ong J.J., Teyeb A., Gaisford S., Campos-Álvarez A., Stulz A., Marcuta C., Kraschew L., Mohr W. (2023). A case study on decentralized manufacturing of 3D printed medicines. Int. J. Pharm. X.

[155] Trenfield S.J., Xu X., Goyanes A., Rowland M., Wilsdon D., Gaisford S., Basit A.W. (2023). Releasing fast and slow: Non-destructive prediction of density and drug release from SLS 3D printed tablets using NIR spectroscopy. Int. J. Pharm. X.

[156] Bendicho-Lavilla C., Rodríguez-Pombo L., Januskaite P., Rial C., Alvarez-Lorenzo C., Basit A.W., Goyanes A. (2024). Ensuring the quality of 3D printed medicines: Integrating a balance into a pharmaceutical printer for in-line uniformity of mass testing. J. Drug Deliv. Sci. Technol..

[157] Salunke S., Agrawal A., Walsh J., Nunn A., Hughes K., Kuehl P., Caivano G., Clapham D., Thompson K., Rumondor A. (2024). Selecting appropriate excipients for paediatric dosage form − Paediatric excipients risk assessment (PERA) framework—Part 1. Eur. J. Pharm. Biopharm..

[158] Agrawal A., Salunke S., Rumondor A., Thompson K., Caivano G., Walsh J., Enright B., Sherratt P., Hughes K., Clapham D. (2024). Paediatric excipient risk assessment (PERA) tool and application for selecting appropriate excipients for paediatric dosage forms—Part 2. Eur. J. Pharm. Biopharm..

[159] Elbadawi M., Muñiz Castro B., Gavins F.K.H., Ong J.J., Gaisford S., Pérez G., Basit A.W., Cabalar P., Goyanes A. (2020). M3DISEEN: A novel machine learning approach for predicting the 3D printability of medicines. Int. J. Pharm..

[160] FDA Distributed Manufacturing and Point-of-Care Manufacturing of Drugs. https://www.fda.gov/media/162157/download?attachment.

[161] DRAFT—EMA Regulatory Science Strategy to 2025. https://www.ema.europa.eu/system/files/documents/regulatory-procedural-guideline/ema_regulatory_science_to_2025_en.pdf.

